# Design, Synthesis, and Biological Evaluation of Benzo[cd]indol-2(1H)-ones Derivatives as a Lysosome-Targeted Anti-metastatic Agent

**DOI:** 10.3389/fonc.2021.733589

**Published:** 2021-08-27

**Authors:** Jinghua Li, Shuai Chen, Yancong Zhao, Huiyuan Gong, Tong Wang, Xiaoling Ge, Yuxia Wang, Chenguang Zhu, Liang Chen, Fujun Dai, Songqiang Xie, Chaojie Wang, Wen Luo

**Affiliations:** ^1^Key Laboratory of Natural Medicine and Immuno-Engineering, Henan University, Kaifeng, China; ^2^The First Affiliated Hospital, Henan University, Kaifeng, China; ^3^Institute of Chemical Biology, School of Pharmacy, Henan University, Kaifeng, China; ^4^College of Chemistry and Chemical Engineering Henan University, Kaifeng, China

**Keywords:** polyamine, benzo[cd]indol-2(1H)-ones derivatives, apoptosis, autophagy, metastasis

## Abstract

Lysosomes have become a hot topic in tumor therapy; targeting the lysosome is therefore a promising strategy in cancer therapy. Based on our previous lysosome-targeted bio-imaging agent, homospermine-benzo[cd]indol-2(1H)-one conjugate (HBC), we further developed three novel series of polyamine- benzo[cd]indol-2(1H)-one conjugates. Among them, compound 15f showed potent inhibitory activity in hepatocellular carcinoma migration both *in vitro* and *in vivo*. Our study results showed that compound 15f entered the cancer cells *via* the polyamine transporter localized in the lysosomes and caused autophagy and apoptosis. The mechanism of action revealed that the crosstalk between autophagy and apoptosis induced by 15f was mutually reinforcing patterns. Besides, 15f also targeted lysosomes and exhibited stronger green fluorescence than HBC, which indicated its potential as an imaging agent. To summarize, compound 15f could be used as a valuable dual-functional lead compound for future development against liver-cancer metastasis and lysosome imaging.

## Introduction

Hepatocellular carcinoma (HCC) is one of the leading causes of cancer deaths worldwide owing to its high recurrence rate and poor prognosis (1–3). Since HCC exhibits no identifiable symptoms at the premorbid stage, a majority of patients with liver cancer are often diagnosed at an advanced stage, which is often accompanied with micro-metastases. In such cases, a surgical resection is clearly not feasible; therefore, chemotherapy treatment often always is the main choice to prolong the life of the patient. Therefore, there is an urgent requirement to develop anticancer agents with diagnostic and therapeutic functions in the management of HCC (4).

Autophagy is a multistep lysosomal-degradation pathway. Although a dual role for autophagy in cancer therapy has been previously reported in protecting against and promoting cell death, the potential for using autophagy in cancer therapy appears to be promising (5–10). For example, autophagy inhibitor compounds, including derivatives of chloroquine (CQ) (DC661 and DQ661), exert anticancer effects through targeting PPT1 (11, 12). Besides, it has been reported that several drugs that induce autophagy also demonstrate potent anticancer activity (13, 14).

**Figure 1:**
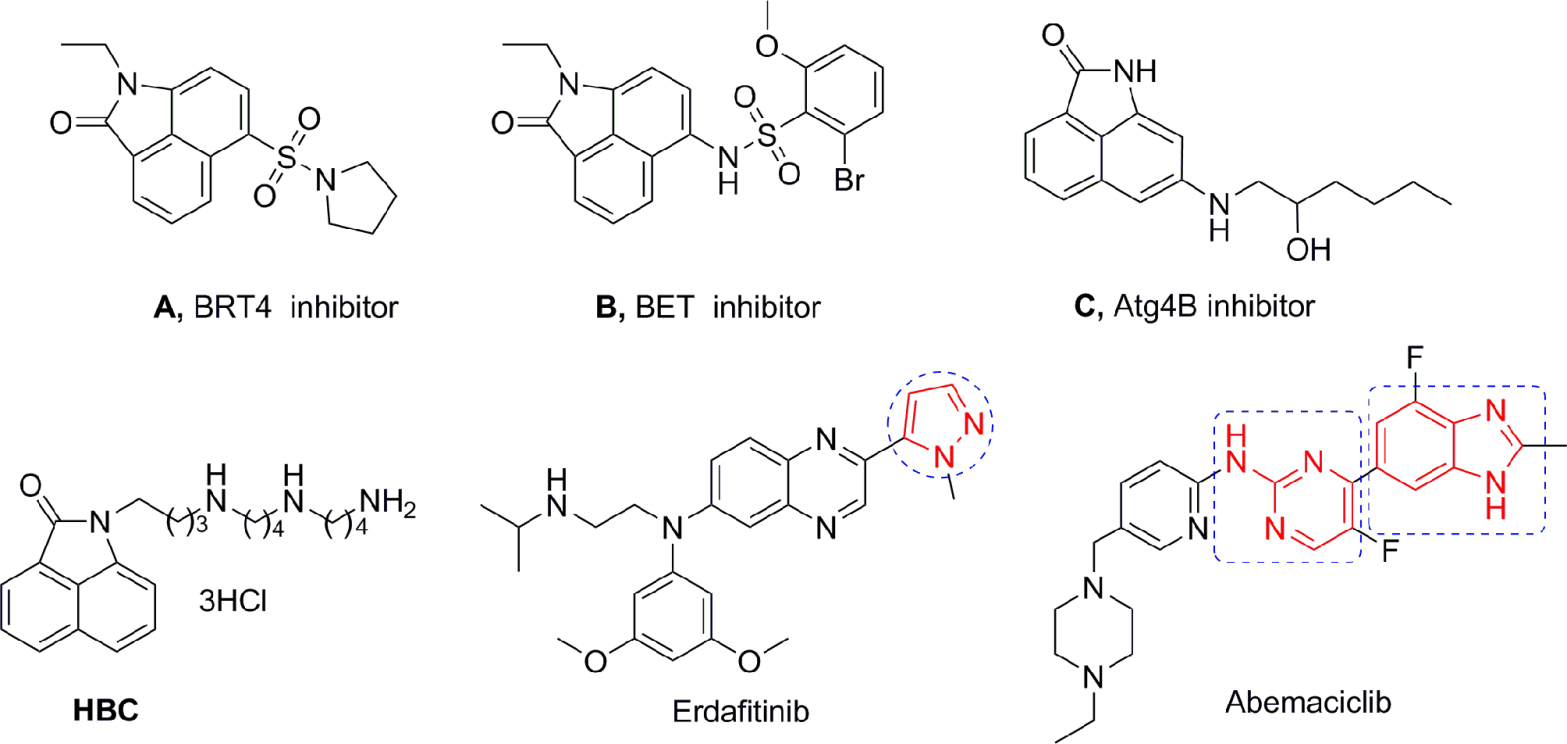
Structures of BIOs, HBC, and marketed heterocyclic drugs (Erdafitinib and Abemaciclib).

Benzo[cd]indol-2(1H)-one (BIO) was originally used in dyes and the manufacture of electronic typing materials. Of late, it is being frequently used as a significant scaffold in a wide range of pharmaceuticals and biologically active compounds (15). Feng et al. designed and synthesized a series of BIOs as BRD4 inhibitors (16), while Xue et al. discovered some BIOs to be BET bromodomain inhibitors (17). The Triola group reported several BIOs as autophagy-related gene Atg4B inhibitors that are being considered as a novel chemotype for further development (**Figure 1**) (18).

Our research group has focused on polyamine conjugates for several years; we reported that the antitumor effects of polyamine conjugates were exerted by the induction of autophagy and apoptosis, and found there was a crosstalk between autophagy and apoptosis (19, 20). Recently, we also reported that the homospermine-BIO conjugate (HBC) (**Figure 1**) could serve as a lysosome-targeting fluorescent probe as well as an antimetastatic agent (21). However, these compounds were only modified using polyamines at the N atom and para position on the BIO scaffold and did not involve other substitutions, expansions, or modifications on the parent structure. Aromatic heterocycle compounds play a major role in drug discovery owing to their diverse biological activities (22). Therefore, on the basis of our previous research, herein, in order to expand the diversity of the substituents and scaffold, and to establish a more comprehensive structure-activity relationship (SAR), we selected several amino side chains and para-heterocyclic substitutions (pyrazol, aminopyrimidine, and benzimidazole) to construct three different types of scaffolds. The aim of our study was to explore the chemistry and activity of these derivatives as potential drugs to determine their use as fluorescent probes for subcellular organelles, and to conduct an in-depth SAR investigation. The newly synthesized compounds were evaluated for anticancer activities. *In vitro* and *in vivo* assays showed that compound 15f targeted the lysosomes and inhibited pulmonary metastasis by inducing apoptosis and autophagy through lysosome-mediated pathways.

## Experimental Section

### Chemistry

All solvents and reagents were purchased from the suppliers and used without further purification. Melting points were determined using an X-6 hot stage microscope and were not corrected. ^1^H and ^13^C NMR spectra were recorded in D_2_O (CDCl_3_ or DMSO-*d*_6_) with a Bruker AV-300 spectrometer. ESI-MS spectra were recorded on an ESQUIRE-LC mass spectrometer. Elemental analyses were performed on a Gmbe VarioEL elemental instrument, and results were within 0.4% of the theoretical values.

### General Procedure for the Synthesis of Compounds 9a,9b, 9d, 9g, 10a, 10b, 10d, and 10g

Acetyl chloride (1.86 g, 23.64 mmol) was quickly added to a solution of 1 (1.99 g, 11.82 mmol), AlCl_3_ (7.88 g, 59.1 mmol) in CS_2_ (20 mL) under ice-bath. The mixture was stirred at 0°C for 3 h. Then the ice-bath was removed, and the mixture was heated at 45°C for 12 h. After cooling to room temperature, the mixture was diluted with ice water (30 ml) and extracted with EtOAc, dried over MgSO_4_, and concentrated under reduced pressure. The residue was purified by column chromatography (petroleum ether/EtOAc = 4:1) to afford the intermediate 2.

6-acetylbenzo[cd]indol-2(1H)-one (**2**), yield 82%; ^1^H NMR (CDCl_3_, 300 MHz) *δ* 9.20 (d, J =8.43 Hz,1H), 8.72 (s, 1H), 8.12 (t, J=8.17 Hz, 2H), 7.84 (t, J=7.74 Hz, 1H), 7.02 (d, J=7.56 Hz,1H), 2.72 (s, 3H, 1×CH_3_); ^13^C NMR (CDCl_3_, 75 MHz) *δ* 198.05, 168.53, 140.56, 133.48, 131.77, 129.53, 127.23, 126.56, 125.88, 124.82, 124.00, 103.91, 27.03; ESI-MS m/z: 212.08 [M+H]^+^.

To a solution of 2 (1.01 g, 5.1 mmol) and anhydrous K_2_CO_3_ (2.11 g, 15.3 mmol) in 15 ml of CH_3_CN was added 1,4-dibromobutane (1.35 g, 6.27 mmol). The mixture was refluxed for 5 h and concentrated. Then the residue was dissolved in CH_2_Cl_2_ and washed with aqueous Na_2_CO_3_. The organic layer was dried with Na_2_SO_4_ and concentrated. The residue was purified by column chromatography (petroleum ether/EtOAc = 2:1) to give the intermediate 3.

6-acetyl-1-(4-bromobutyl)benzo[cd]indol-2(1H)-one (3), yield 75%; ^1^H NMR (CDCl_3_, 300 MHz) *δ* 9.11 (d, J=8.40 Hz, 1H), 8.09 (d, J=7.62 Hz, 1H), 8.01 (d, J=7.02 Hz, 1H), 7.77 (t, J=6.86 Hz, 1H), 6.87 (d, J=7.61 Hz, 1H), 3.92 (t, J=3.96 Hz, 2H), 3.44 (t, J=2.38 Hz, 2H), 2.69 (s, 3H, 1×CH_3_), 1.97-1.88 (m, 4H); ^13^C NMR (CDCl_3_, 75 MHz) *δ* 198.87, 168.23, 143.70, 134.53, 132.52, 130.67, 127.96, 127.17, 125.56, 124.89, 103.34, 39.15, 33.09, 29.67, 28.16, 27.17; ESI-MS m/z: 346.01 [M+H]^+^.

Intermediate 3 (1.23 g, 3.57 mmol), piperidine (4.28 mmol), and anhydrous K_2_CO_3_ (0.89 g, 6.43 mmol) were mixed in dry CH_3_CN and refluxed for 8 h. After cooling, the mixture was concentrated and dissolved in EtOAc, washed by aqueous Na_2_CO_3_, dried over Na_2_SO_4_, and concentrated in vacuo. The residue was purified by column chromatography (CH_2_Cl_2_/MeOH = 30:1) to give 5d as yellow oil.

Intermediate 3 (1.23 g, 3.57 mmol), amines (4.28 mmol), and anhydrous K_2_CO_3_ (0.89 g, 6.43 mmol) were mixed in dry CH_3_CN (25 mL), and the reaction mixture was refluxed for 8 h. After cooling, the mixture was concentrated in vacuum and dissolved in EtOAc (20 ml). The organic layer was washed by aqueous Na_2_CO_3_, dried over Na_2_SO_4_, concentrated, and the residue was diluted with MeOH (30 ml), then triethylamine (8.6 mmol) and (Boc)_2_O (0.97 g, 9.56 mmol) were added and the mixture was further stirred at room temperature overnight. Then the solvent was concentrated in vacuo, and the residue was poured into H_2_O, extracted with EtOAc, washed by aqueous Na_2_CO_3_, dried over anhydrous Na_2_SO_4_, and then evaporated and purified by flash column chromatography (petroleum ether/EtOAc = 2:1) to produce 5a, 5b, 5g.

A mixture of 5 (2.5 mmol) and DMF-DMA (1.19 g, 10 mmol) were dissolved in DMF (15 ml), then stirred at 90°C for 8 h. The mixture was concentrated in vacuo and re-dissolved in EtOAc (20 ml). The organic layer was washed with aqueous Na_2_CO_3_, dried over Na_2_SO_4_, and concentrated in vacuo. The residue was purified by column chromatography (CH_2_Cl_2_/MeOH = 20:1) to obtain 6a, 6b, 6d, 6g as a yellow oil.

(1-(4-(1-piperidyl)butyl)-6-[(3-dimethylamino)acrylyl]benzo[cd]indol-2(1H)-one (6d), yield 55%; ^1^H NMR (CDCl_3,_ 300 MHz) *δ* 8.88-8.70 (m, 1H), 8.01 (d, J=6.93 Hz, 1H), 7.86-7.78 (m, 1H), 7.76-7.67 (m, 2H), 6.86 (d, J=7.50 Hz, 1H), 5.70-5.55 (m, 1H), 3.90 (t, J=7.21 Hz, 2H), 3.11 (s, 2H), 2.90 (m, 4H), 2.29 (t, J=7.43 Hz, 6H), 1.77 (m, 2H), 1.61-1.49 (m, 6H), 1.38-1.40 (m, 2H); ^13^C NMR (CDCl_3,_ 75 MHz) *δ* 192.86, 190.12, 168.29, 154.14, 150.81, 141.31, 133.03, 131.57, 129.82, 129.51, 127.38, 126.19, 124.40, 103.90, 95.68, 94.83, 58.80, 58.77, 54.54, 40.07, 26.83, 25.92, 24.39, 24.24; ESI-MS m/z: 406.13 [M+H]^+^.

{1-[4-(3-diethylaminopropyl)butoxycarbonylaminobutyl]}-6-[(3-dimethylamino)acrylyl]benzo[cd]indol-2(1H)-one (6a), yield 40%; ^1^H NMR (CDCl_3_, 300 MHz) *δ* 8.77 (d, J=8.40 Hz, 1H), 8.05 (d, J =7.04 Hz, 1H), 7.84 (d, J=7.47 Hz, 1H), 7.79-7.69 (m, 2H), 6.91 (s, 1H), 5.64 (d, J=6.84 Hz, 1H), 3.95 (t, J=7.06 Hz, 2H), 3.05 (d, J=5.97 Hz, 10H), 2.50 (m, 6H), 1.82-1.59 (m, 6H), 1.43 (s, 9H), 1.03-0.96 (m, 6H); ^13^C NMR (CDCl_3_,75 MHz) *δ* 155.59, 154.19, 141.19, 133.15, 131.68, 129.49, 129.31, 126.09, 124.49, 103.94, 95.61, 79.23, 66.87, 58.62, 53.62, 39.86, 28.45, 25.98, 23.69; ESI-MS m/z: 551.22 [M+1]^+^.

{1-[4-(4-morpholinobutyl)butoxycarbonylaminobutyl]}-6-[(3-dimethylamino)acrylyl]benzo[cd]indol-2(1H)-one (6b), yield 54%; ^1^H NMR (CDCl_3_, 300 MHz) *δ* 8.42 (m, 1H), 7.65 (d, J=6.43 Hz, 1H), 7.40 (t, J=7.84Hz, 2H), 6.57 (t, J=8.05 Hz, 2H), 5.81 (s, 1H), 3.59 (d, J=8.71 Hz, 2H), 3.34 (s, 4H), 2.76-2.69 (m, 10H), 1.99 (d, J=3.42 Hz, 6H), 1.44-1.39 (m, 2H), 1.28 (m, 2H), 1.14-1.10 (m, 13H); ^13^C NMR (DMSO, 75 MHz) *δ* 193.40, 167.90, 155.98, 155.09, 145.06, 141.82, 131.85, 130.44, 126.80, 125.98, 125.71, 125.55, 105.55, 78.72, 77.89, 46.78, 44.74, 38.02, 28.67, 28.36, 25.89; ESI-MS m/z: 579.29 [M+H]^+^.

{1-[4-(4-(4-butoxycarbonylaminobutyl)butoxycarbonylaminobutyl)butoxycarbonyl aminobutyl]}-6-[(3-dimethylamino)acrylyl]benzo[cd]indol-2(1H)-one (6g), yield 54%; ^1^H NMR (CDCl_3_, 300 MHz) *δ* 8.55 (d, J=8.31 Hz, 1H), 7.78 (d, J=7.06 Hz, 1H), 7.61 (d, J=7.42 Hz, 1H), 7.57-7.46 (m, 2H), 6.68 (t, J=8.6.4 Hz, 1H), 5.42 (d, J=8.14 Hz, 1H), 3.70 (t, J=7.12 Hz, 2H), 3.12 -2.63 (m, 16H), 1.55 (s, 2H), 1.43-1.09 (m, 37H); ^13^C NMR (CDCl_3_,75 MHz) *δ* 167.95, 156.08, 155.64, 151.14, 140.42, 129.39, 126.77, 125.87, 124.58, 122.74, 104.19, 79.42, 79.16, 77.28, 46.57, 40.20, 39.81, 28.49, 28.43, 27.36, 25.94; ESI-MS m/z: 780.32 [M+H]^+^.

To a solution of 6 (1.7 mmol) in EtOH (15 ml) was added hydrazine hydrate (0.26 g, 5.1 mmol). The mixture was refluxed for 10 h, then cooled to room temperature and concentrated in vacuo. The residue was extracted with CH_2_Cl_2_. The combined organic phases were washed with aqueous Na_2_CO_3_, dried over NaSO_4_, and concentrated in vacuo. The residue was purified by column chromatography (CH_2_Cl_2_/MeOH = 10:1) to obtain 7a, 7b, 7d, 7g as yellow oil.

Intermediates 7a, 7b, 7d, and 7g were dissolved in EtOH (10 ml) and stirred at 0°C for 10 min. Then 4 mol/L HCl was added dropwise at 0°C. The reaction mixture was stirred at room temperature overnight. The solution typically gave a white solid as precipitate, filtered, washed several times with absolute ethanol, and dried under vacuum to give the pure target compounds 9a, 9b, 9d, and 9g.

(1-(4-(1-piperidyl)butyl)-[6-(1H-pyrazol-5-yl)]benzo[cd]indol-2(1H)-one dihydrochloride (9d), mp 207.8-210.0°C; yield 49%; ^1^H NMR (D_2_O, 300 MHz) *δ* 7.67 (t, J=4.92 Hz, 2H), 7.20 (d, J=3.29 Hz, 1H), 7.02 (t, J=6.34 Hz, 1H), 6.95 (d, J=5.24 Hz, 1H), 6.33 (d, J=6.21 Hz, 1H), 6.22 (d, J=3.89 Hz, 1H), 3.28 (d, J=4.63 Hz, 2H), 3.17 (t, J=6.12 Hz, 2H), 2.79 (t, J=6.24 Hz, 2H), 2.66-2.64 (m, 2H), 1.83-1.15 (m, 10H); ^13^C NMR (D_2_O, 75 MHz) *δ* 168.61, 146.72, 136.59, 132.79, 129.97, 128.51, 127.37, 125.13, 124.16, 123.79, 122.99, 106.59, 105.04, 55.96, 52.98, 38.86, 24.85, 22.67, 21.00, 20.61. ESI-MS m/z: 375.12 [M+H]^+^; Elemental Analysis for C_23_H_28_Cl_2_N_4_O: C 61.74, H 6.31, N 12.52; found: C 61.52, H 6.70, N 12.86.

{1-[4-(3-diethylaminopropyl)aminobutyl]}-[6-(1H-pyrazol-5-yl)]benzo[cd]indol-2(1H)-one trihydrochloride (9a), mp 117.7-120.1°C; yield: 40%. ^1^H NMR (D_2_O, 300 MHz) *δ* 7.93 (s, 1H), 7.59 (d, J=7.50 Hz, 1H), 7.35 (d, J=3.86 Hz, 1H), 7.14 (d, J=6.28 Hz, 2H), 6.60 (d, J=6.16 Hz, 1H), 6.49 (s, 1H), 3.44 (d, J=6.60 Hz, 2H), 3.24-3.19 (m, 6H), 3.09 (t, J=6.78 Hz, 2H), 3.00(t, J=6.84 Hz, 2H), 2.10-2.08 (m, 2H), 1.62-1.50 (m, 4H), 1.26 (t, J=6.42 Hz, 6H); ^13^C NMR (D_2_O, 75 MHz) *δ* 168.63, 145.20, 137.94, 133.57, 129.10, 128.99, 128.69, 124.66, 124.40, 123.98, 120.28, 106.52, 106.09, 48.32, 47.43, 47.15, 44.28, 39.18, 24.92, 22.87, 20.64, 8.14. ESI-MS m/z: 420.18 [M+H]^+^; Elemental Analysis for C_25_H_36_Cl_3_N_5_O: C 56.77, H 6.86, N 13.24; found: C 56.72, H 6.47, N 13.30.

{1-[4-(4-morpholinobutyl)aminobutyl]}-[6-(1H-pyrazol-5-yl)]benzo[cd]indol-2(1H)-one trihydrochloride (9b), mp 150.2-153.4°C; yield 42%; ^1^H NMR (D_2_O, 300 MHz) *δ* 7.92 (d, J=3.24 Hz, 1H), 7.73 (d, J=8.31 Hz, 1H), 7.43 (d, J=6.69 Hz, 1H), 7.26-7.20 (m, 2H), 6.65 (d, J=7.44 Hz, 1H), 6.54 (d, J=2.15 Hz, 1H), 4.05 (d, J=10.92 Hz, 2H), 3.74(t, J=12.03 Hz, 2H), 3.46 (t, J=6.42Hz, 4H), 3.15-3.05 (m, 4H), 2.99~2.90 (m, 4H), 1.74-1.54 (m, 8H); ^13^C NMR (D_2_O, 75 MHz) *δ* 168.84, 145.55, 137.96, 133.43, 129.30, 129.15, 128.70, 124.91, 124.54, 124.17, 120.84, 106.65, 106.05, 63.66, 56.16, 51.56, 46.89, 46.53, 39.20, 24.89, 22.83, 22.56, 20.29. ESI-MS m/z: 448.13 [M+H]^+^; Elemental Analysis for C_26_H_36_Cl_3_N_5_O_2_: C 56.07, H 6.52, N 12.57, found: C 55. 85, H 6.91, N 12.39.

{1-[4-4-(4-aminobutyl)aminobutylaminobutyl]}-[6-(1H-pyrazol-5-yl)]benzo[cd]indol-2(1H)-one tetrahydrochloride (9g), mp 193.5-196.8°C; yield 42%; ^1^H NMR (D_2_O, 300 MHz) *δ* 7.86 (d, J=3.24 Hz, 1H), 7.77 (t, J=8.16 Hz, 1H), 7.41 (t, J=6.92 Hz, 1H), 7.25-7.16 (m, 2H), 6.60 (d, J=3.38 Hz, 1H), 6.48 (d, J=3.84 Hz, 1H), 3.45 (s, 2H), 3.04-2.95 (m, 10H), 1.78-1.72 (m, 8H), 1.56-1.53 (m, 4H); ^13^C NMR (D_2_O, 75 MHz) *δ* 168.80, 146.01, 137.34, 133.22, 129.58, 128.85, 128.10, 124.37, 124.06, 122.07, 106.70, 105.62, 46.94, 46.84, 46.78, 46.68, 39.15, 38.71, 24.89, 23.85, 22.88, 22.72, 22.70; ESI-MS m/z: 449.19 [M+H]^+^; Elemental Analysis for C_26_H_40_Cl_4_N_6_O: C 52.53, H 6.78, N 14.14; found: C 52.35, H 7.01, N 13.95.

To a solution of **6** (1.7 mmol) in EtOH (15 ml) was added 1,5-diazabicyclo(5,4,0) udnec-5-ene (DBU) (0.52 g, 3.4 mmol) and guanidine hydrochloride (0.49 g, 5.1 mmol). The mixture was refluxed for 24 h, then cooled to room temperature and concentrated in vacuo. The residue was dissolved in CH_2_Cl_2_ (30_ ml_) and washed with aqueous Na_2_CO_3_, dried over Na_2_SO_4_, and evaporated. The residue was purified by column chromatography (CH_2_Cl_2_/MeOH = 10:1) to obtain 8a, 8b, 8d, 8g as yellow-green oil.

The target compounds 10a, 10b, 10d, 10g were obtained from 8a, 8b, 8d, 8g according to the same procedure of 9a, 9b, 9d, 9g.

{1-[4-(1-piperidyl)butyl]}-[6-(2-aminopyrimidin-4-yl)]benzo[cd]indol-2(1H)-one dihydrochloride (10d), mp 168.6-171.2°C; yield 41%; ^1^H NMR (D_2_O, 300 MHz) *δ* 8.41 (d, J=8.43Hz, 1H), 8.08 (d, J=6.75Hz, 1H), 7.64 (d, J=7.74Hz, 1H), 7.56 (d, J=6.93Hz, 1H), 7.38 (t, J=7.74Hz, 1H), 7.09 (d, J=6.84 Hz, 1H), 6.74 (d, J=7.71Hz, 1H), 3.57(t, J=5.20Hz, 2H), 3.42 (d, J=10.31Hz, 2H), 3.03 (t, J=8.04Hz, 2H), 2.83(t, J=10.02, 2H), 1.88-1.83(m, 2H), 1.76-1.60 (m, 7H), 1.45-1.37 (m, 1H); ^13^C NMR (D_2_O, 75 MHz) *δ* 171.76, 169.27, 154.77, 145.66, 141.94, 134.11, 131.78, 130.24, 125.58, 125.15, 125.03, 124.13, 108.23, 106.23, 56.13, 53.10, 39.25, 25.10, 22.74, 21.05, 20.79; ESI-MS m/z: 402.13 [M+H]^+^; Elemental Analysis for C_24_H_29_Cl_2_N_5_O: C 60.76, H 6.16, N 14.76; found: C 60.56, H 6.42, N 14.95.

{1-[4-(3-diethylaminopropyl)aminobutyl]}-[6-(2-aminopyrimidin-4-yl)]benzo[cd]indol-2(1H)-one trihydrochloride (10a), mp 148.8-151.9°C; yield 35%; ^1^H NMR (D_2_O, 300 MHz) *δ* 8.50 (d, J=4.29 Hz, 1H), 8.14 (d, J=6.04 Hz, 1H), 7.73 (d, J=5.29 Hz, 1H), 7.68 (d, J=4.26 Hz, 1H), 7.47-7.48 (m, 1H), 7.15 (d, J=6.04 Hz, 1H), 6.85 (d, J=5.18 Hz, 1H), 3.67 (s, 2H), 3.29-3.15 (m, 6H), 3.08 (t, J=6.27 Hz, 4H), 2.15-2.00 (m, 2H), 1.69 (d, J=6.17 Hz, 4H), 1.25 (t, J=6.81Hz, 6H); ^13^C NMR (D_2_O, 75 MHz) *δ* 169.41, 154.80, 145.73, 141.98, 134.12, 131.78, 130.26, 125.62, 125.21, 124.25, 124.02, 108.34, 106.35, 48.30, 47.44, 47.21, 44.29, 39.35, 25.05, 22.92, 20.61, 8.13. ESI-MS m/z: 447.19[M+H]^+^; Elemental Analysis for C_26_H_37_Cl_3_N_6_O: C 56.17, H 6.71, N 15.12; found: C 56.37, H 7.02, N 15.10.

{1-[4-(4-morpholinobutyl)aminobutyl]}-[6-(2-aminopyrimidin-4-yl))]benzo[cd]indol-2(1H) -one trihydrochloride (10b), mp 155.6-158.3°C; yield 39%; ^1^H NMR (D_2_O, 300 MHz) *δ* 8.58 (d, J=8.49 Hz, 1H), 8.19(d, J=6.36 Hz, 1H), 7.81-7.78(m, 2H), 7.58 (d, J=7.62 Hz, 1H), 7.22(d, J=6.78 Hz, 1H), 6.94 (d, J=7.64 Hz, 1H), 4.10-4.05 (m, 2H), 3.81-3.73 (m, 4H), 3.48 (d, J=9.02 Hz, 2H), 3.18-3.14(m, 4H), 3.04-2.99 (m, 4H), 1.78-1.70 (m, 8H); ^13^C NMR (D_2_O, 75 MHz) *δ* 171.82, 169.38, 145.67, 141.99, 134.14, 131.80, 130.26, 125.61, 125.21, 125.12, 124.23, 124.00, 108.30, 106.34, 63.72, 56.22, 51.63, 47.03, 46.65, 39.38, 25.06, 22.94, 22.63, 20.34. ESI-MS m/z: 475.18 [M+H]^+^; Elemental Analysis for C_27_H_37_Cl_3_N_6_O_2_: C 55.53, H 6.39, N 14.39; found: C 55.59, H 6.72, N 14.44.

{1-[4-(4-aminobutyl)aminobutylaminobutyl]}-[6-(2-amino-pyrimidin-4-yl)]benzo[cd]indol -2(1H)-one tetrahydrochloride (10g), mp 249.2-252.5°C; yield 36%; ^1^H NMR (D_2_O, 300 MHz) *δ* 8.41 (d, J=7.18 Hz, 1H), 8.05 (d, J=6.02 Hz, 1H), 7.64 (d, J=4.24 Hz, 1H), 7.56 (d, J=5.16 Hz, 1H), 7.37 (t, J=5.26 Hz, 1H), 7.08 (d, J=4.89 Hz, 1H), 6.75 (d, J=5.46 Hz, 1H), 3.56 (t, J=4.88 Hz, 2H), 2.98-3.01(m, 10H), 1.70-1.62 (m, 12H); ^13^C NMR (D_2_O, 75 MHz) *δ* 171.89, 169.18, 144.75, 141.99, 134.31, 131.90, 130.21, 125.46, 125.08, 124.54, 123.94, 107.84, 106.23, 57.29, 47.01, 46.81, 46.75, 39.30, 38.65, 25.03, 23.81, 22.93, 22.71, 22.67, 16.64; ESI-MS m/z: 476.22[M+H]^+^; Elemental Analysis for C_27_H_41_Cl_4_N_7_O: C 52.18, H 6.65, N 15.78; found: C 51.89, H 6.72, N 15.52.

### General Procedure for the Synthesis of Compounds 15c-15h

Intermediate 11 was prepared by a procedure reported previously (18). DMF (15 ml) was added to a solution of POCl_3_ (15 ml) gradually with stirring and cooling. After the reaction mixture stirring at 0°C for 2 h, a solution of compound 11 (6 g, 18.06 mmol) in 5 ml DMF was added dropwise, and then the mixture was heated at 50°C for 72 h. After completion, the mixture was poured into ice water and extracted with CH_2_Cl_2_ three times. The organic phases were washed with brine, dried over Na_2_SO_4_, and concentrated. The residue was purified by flash column chromatography (petroleum ether/ethyl acetate = 1:1) to obtain compound 12 as yellow solid.

6-formyl-1-(4-bromobutyl)benzo[cd]indol-2(1H)-one (12), yield 60%; ^1^H NMR (CDCl_3_, 300 MHz) *δ* 10.16(s, 1H), 9.12(d, J=8.01 Hz, 1H), 8.07(d, J=7.02Hz, 1H), 7.96(d, J=7.41Hz, 1H), 7.83(m, 1H), 7.01(d, J=7.42Hz, 1H), 3.97(t, J=6.74 Hz, 2H), 3.60(t, J=6.14 Hz, 2H), 2.02-1.84(m, 4H); ^13^C NMR (CDCl_3,_ 75 MHz) *δ* 192.09, 168.30, 144.99, 140.69, 131.18, 131.01, 127.25, 126.33, 125.48, 103.86, 44.34, 39.45, 29.59, 25.96; ESI-MS m/z: 332.12[M+H]^+^.

Compound 12 (1.32 g, 4 mmol), K_2_CO_3_ (1.48 g, 10.71 mmol), and amines (4.8 mmol) were mixed in dry CH_3_CN (30 ml). The reaction mixture was refluxed for 8 h. After cooling, the reaction mixture was concentrated under reduced pressure. The residue was diluted with CH_2_Cl_2_, washed with aqueous Na_2_CO_3_, dried over Na_2_SO_4_, and concentrated. The residues were further purified by column chromatography (CH_2_Cl_2_/MeOH = 30:1) to obtain the intermediates 13d-13e as yellow oil.

Compound 12 (1.99 g, 6 mmol), K_2_CO_3_ (0.99 g, 7.2 mmol), and polyamine (7.2 mmol) were mixed in dry CH_3_CN (30 ml). The reaction mixture was refluxed for 8 h. After cooling, the reaction mixture is concentrated under reduced pressure. The residue was diluted with CH_3_OH (30 ml), then Boc_2_O (2.36 g, 10.8 mmol) and triethylamine (0.73 g, 7.2 mmol) were added and stirred at room temperature overnight. The mixture was evaporated in vacuo, and the residue was diluted with H_2_O (30 ml) and extracted with CH_2_Cl_2_ (30 ml), washed by aqueous Na_2_CO_3_, dried over Na_2_SO_4_, and evaporated in vacuo. The residue was purified by column chromatography (petroleum ether/EtOAc = 2:1) to obtain the intermediates 13c, 13g, 13f, 13h as yellow oil.

A mixture of 13 (2 mmol) and o-phenylenediamine (0.26 g, 2.4 mmol) in DMF (10 ml) was refluxed for 12 h. The mixture was diluted with H_2_O (10 ml) and extracted with EtOAc three times. The organic phases were washed with brine, dried over Na_2_SO_4_, and evaporated. The residue was purified by flash column chromatography (petroleum ether/EtOAc = 1:1) to obtain 14c-14h.

1-(4-((4-aminobutyl)amino)butyl)-6-(1H-benzo[d]imidazol-2-yl)benzo[cd]indol-2(1H)-one (14h); yield 64%, ^1^H NMR (CDCl_3_, 300 MHz) *δ* 8.66 (d, J=8.56 Hz, 1H), 7.91-7.25 (m, 7H), 6.39 (d, J=6.39 Hz, 1H), 3.74 (s, 2H), 3.28-2.82 (m, 6H), 1.63-1.24 (m, 26H); ^13^C NMR(CDCl_3_, 75 MHz) *δ* 167.83, 156.19, 155.68, 151.24, 140.05, 131.03, 129.41, 128.91, 126.61, 125.64, 124.76, 124.42, 122.64, 121.41, 104.11, 79.46, 79.17, 46.77, 40.15, 39.65, 28.44, 28.40, 27.27, 25.88; ESI-MS m/z: 628.28 [M+H]^+^.

6-(1H-benzo[d]imidazol-2-yl)-1-(4-((4-(cyclopropylamino)butyl)amino)butyl)benzo[cd]indol-2(1H)-one (14c), yield 48%; ^1^H NMR (CDCl_3_, 300 MHz) *δ* 8.09-7.51 (m, 6H), 7.36 -7.28 (m, 2H), 6.63 (s, 1H), 3.85(s, 2H), 3.14 (d, J=6.06 Hz, 6H), 2.37 (s, 1H), 1.80-1.30 (m, 30H); ^13^C NMR (CDCl_3_, 75 MHz) *δ* 167.90, 156.93, 150.77, 140.78, 131.02, 129.76, 129.33, 126.62, 124.67, 123.01, 104.16, 79.58, 47.14, 39.86, 28.51, 28.45, 26.08, 8.13; ESI-MS m/z: 668.32[M+H]^+^.

1-(4-((4-((4-aminobutyl)amino)butyl)amino)butyl)-6-(1H-benzo[d]imidazol-2-yl)benzo[cd]indol-2(1H)-one (14g), yield 55%; ^1^H NMR (CDCl_3_, 300 MHz) *δ* 8.82 (d, J=8.01 Hz, 1H), 8.06-7.21 (m, 7H), 6.54 (s, 1H), 3.81 (t, J=6.92 Hz, 2H), 3.15 (d, J=6.29 Hz, 10H), 1.84~1.27 (m, 39H); ESI-MS m/z: 799.45[M+H]^+^.

1-(4-((3-((4-aminobutyl)amino)propyl)amino)butyl)-6-(1H-benzo[d]imidazol-2-yl)benzo[cd]indol-2(1H)-one (14f), yield 50%; ^1^H NMR (CDCl_3_, 300 MHz) *δ* 8.80 (d, J= 8.13 Hz, 1H), 7.94-7.26 (m, 7H), 6.52 (s, 1H), 3.80 (t, J=6.54 Hz, 2H), 3.29-2.98 (m, 10H), 1.71-1.31 (m, 37H); ESI-MS m/z: 785.42[M+H]^+^.

The target compounds 15c–h were obtained from 14c–h according to the same procedure of 9a,9b,9d,9g.

{1-[4-(1-piperidyl)butyl]}-[6-(1H-benzo[d]imidazol-2-yl)]benzo[cd]indol-2(1H)-one (15d), mp 75.2-78.9°C; yield: 45%; ^1^H NMR (DMSO, 300 MHz) *δ* 12.97 (s, 1H), 9.57 (d, J=8.42 Hz, 1H), 8.22 (d, J=7.26 Hz, 1H), 8.14 (d, J=6.9 Hz, 1H), 7.95 (t, J=7.70 Hz, 1H), 7.75 (br.s, 1H), 7.58 (br.s, 1H), 7.41 (d, J=7.81 Hz, 1H), 7.24 (d, J=4.83 Hz, 2H), 3.92 (t, J=6.45 Hz, 2H), 2.24-2.21 (m, 6H), 1.74-1.72 (m, 2H), 1.47-1.38 (m, 8H); ^13^C NMR (DMSO, 75 MHz) *δ* 167.48, 151.48, 140.88, 132.62, 130.18, 129.83, 127.11, 126.25, 125.29, 125.00, 122.62, 121.10, 119.34, 111.68, 105.96, 58.31, 54.41, 26.51, 26.02, 24.60, 24.06; ESI-MS m/z: 425.11[M+H]^+^; Elemental Analysis for C_27_H_28_N_4_O: C 76.39, H 6.65, N 13.20; found: C76.02, H 6.91, N 12.84.

{1-[4-(1-piperazinyl)butyl]}-[6-(1H-benzo[d]imidazol-2-yl)]benzo[cd]indol-2(1H)-one trihydrochloride (15e), mp 188.2-191.1°C; yield: 45%; ^1^H NMR (D_2_O, 300 MHz) *δ* 7.94 (d, J=6.89 Hz, 1H), 7.83-7.70 (m, 2H), 7.60 (d, J= 8.14 Hz, 1H), 7.30-7.22 (m, 4H), 7.07 (d, J=8.12 Hz, 1H), 3.71-3.65 (m, 10H), 3.35 (t, J=8.15 Hz, 2H), 1.85-1.72 (m, 4H); ^13^C NMR (D_2_O, 75 MHz) *δ* 168.90, 145.96, 142.09, 133.31, 131.24, 130.50, 127.85, 126.31, 126.13, 124.91, 123.59, 113.11, 112.94, 106.44, 56.56, 48.40, 40.68, 39.42, 24.87, 20.71; ESI-MS m/z: 426.11[M+H]^+^; Elemental Analysis for C_26_H_27_N_5_O·3HCl: C 58.38, H 5.65, N 13.09; found: C58.29, H 5.54, N 12.98.

{1-[4-(4-aminobutyl)aminobutyl]}-[6-(1H-benzo[d]imidazol-2-yl)]benzo[cd]indol-2(1H)-one trihydrochloride (15h), mp 186.5-189.4°C; yield 44%; ^1^H NMR (D_2_O, 300 MHz) *δ* 7.82 (d, J=7.21 Hz, 1H), 7.69-7.64 (m, 2H), 7.46 (d, J=7.56 Hz, 1H), 7.21-7.10 (m, 4H), 6.98 (d, J=7.24 Hz, 1H), 3.59 (s, 2H), 3.11-3.09 (m, 6H), 1.79-1.66 (m, 8H); ^13^C NMR (D_2_O, 75 MHz) *δ* 168.57, 145.46, 142.04, 133.29, 131.30, 130.16, 127.55, 126.32, 126.08, 124.71, 112.96, 112.37, 106.39, 47.03, 46.89, 39.60, 38.82, 25.03, 23.93, 23.00, 22.78; ESI-MS m/z: 428.13[M+H]^+^; Elemental Analysis for C_26_H_32_Cl_3_N_5_O: C 58.16, H 6.01, N 13.04; found: C 57.93, H 6.28, N 13.37.

{1-[4-(4-cyclopropylamino)aminobutyl]}-[6-(1H-benzo[d]imidazol-2-yl)]benzo[cd]indol-2(1H)-one trihydrochloride (15c), mp 115.6-118.5°C; yield 33%; ^1^H NMR (D_2_O, 300 MHz) *δ* 7.87 (d, J=7.24 Hz, 1H), 7.78-7.66 (m, 2H), 7.54 (d, J=7.54 Hz, 1H), 7.27-7.18 (m, 4H), 7.03 (d, J= 7.68 Hz, 1H), 3.66 (t, J=7.25 Hz, 2H), 3.24 (t, J=6.31 Hz, 2H), 3.13-3.09 (m, 4H), 2.82-2.74 (m, 1H), 1.83-1.73 (m, 8H), 0.95-0.91 (m, 4H); ^13^C NMR (D_2_O, 75 MHz) *δ* 168.65, 142.14, 133.39, 131.33, 130.18, 127.59, 126.39, 126.12, 124.80, 123.43, 113.01, 112.42, 106.43, 47.34, 47.03, 46.86, 39.62, 29.96, 25.04, 23.01, 22.87, 22.62, 2.95; ESI-MS m/z: 468.18[M+H]^+^; Elemental Analysis for C_29_H_36_Cl_3_N_5_O: C 60.37, H 6.29, N 12.14; found: C 60.76, H 6.66, N 12.43.

{1-[4-(4-(4-aminobutyl)aminobutyl)aminobutyl]}-[6-(1H-benzo[d]imidazol-2-yl)]benzo[cd]indol-2(1H)-one tetrahydrochloride (15g), mp 219.8-222.3°C; yield 40%; ^1^H NMR (D_2_O, 300 MHz) *δ* 8.17 (d, J=8.34 Hz, 1H), 7.97 (d, J=6.84 Hz, 1H), 7.90-7.83 (m, 2H), 7.48-7.46 (m, 4H), 7.23 (d, J=7.24Hz, 1H), 3.86 (s, 2H), 3.13-3.06 (m, 10H), 1.79-1.77(m, 12H); ^13^C NMR (D_2_O, 75 MHz) *δ* 169.07, 146.21, 142.32, 133.43, 131.28, 130.61, 127.97, 126.42, 126.20, 125.12, 123.80, 113.23, 106.55, 47.11, 46.93, 46.87, 39.66, 38.81, 25.06, 23.93, 23.05, 22.82, 22.76; ESI-MS m/z: 499.23[M+H]^+^; Elemental Analysis for C_30_H_42_Cl_4_N_6_O: C 55.91, H 6.57, N 13.04; found: C 55.82, H 6.77, N 13.24.

{1-[4-(3-(4-aminobutyl)aminopropyl)aminobutyl]}-[6-(1H-benzo[d]imidazol-2-yl)] benzo[cd]indol-2(1H)-one tetrahydrochloride (15f), mp 217.8-220.6°C; yield: 48%; ^1^H NMR (D_2_O, 300 MHz) *δ* 7.77 (d, J=6.61, 1H), 7.66-7.61 (m, 2H), 7.44 (d, J=7.51 Hz, 1H), 7.19-7.16 (m, 2H), 7.10-7.08 (m, 2H), 6.95 (d, J=7.51 Hz, 1H), 3.57 (m, 2H), 3.19-3.04 (m, 10H), 2.18-2.08 (m, 2H), 1.78-1.63 (m, 8H); ^13^C NMR (D_2_O, 75 MHz) *δ* 168.55, 145.37, 142.07, 133.37, 131.34, 130.04, 127.48, 126.38, 126.11, 124.70, 123.32, 123.09, 112.94, 112.23, 106.40, 47.22, 47.07, 44.47, 39.59, 38.78, 25.00, 23.88, 23.00, 22.74, 22.71; ESI-MS m/z: 485.20[M+H]^+^; Elemental Analysis for C_29_H_40_Cl_4_N_6_O: C 55.25, H 6.39, N 13.33; found: C 55.37, H 6.71, N 12.68.

### Biological Evaluation

#### Cell Culture and Growth Inhibition Assay

Cells were seeded in 96-well plates at a concentration of 5,000 cells per well. Cells were grown in DMEM (high glucose) containing 10% fetal bovine serum, streptomycin (100 units/ml), and penicillin (100 units/ml). After 24 h, cells were treated with different concentrations of compounds for 24 h, then 50 μl MTT (1 mg/ml) was added and incubated in 37°C for 4 h. After supernatant was removed, DMSO (100 µl) was added to dissolve the crystal product at room temperature for 10 min. The absorbance at 570 nm was measured by a microplate reader. Each experiment was repeated at least three times.

#### Wound Healing Assay

Wound scratch test was carried out as described earlier (23). Cells were grown to confluence and serum-starved overnight. A uniform scratch was placed through the confluent cell monolayer with a pipette tip, and the float cells were washed using PBS. Images of wound area were obtained at 0 and 24 h after scratch. The percentage of closure was calculated as follows: percentage wound closure (%) = distance of migrated cancer cell from the wound edge/total wound width × 100.

#### Cell Migration Assay

Migration assay was determined in 24-well using a Boyden chamber with an 8 μm pore size polycarbonate membrane (Corning, NY, USA). Chambers were washed three times with PBS and then activated in serum-free medium at 37°C. The upper compartment of the chamber was added with 200 μl of serum-free medium (containing 1×10^5^ cells), while the lower compartment was added 600 μl DMEM and 10% FBS. Cells were treated with 15f for 24 h, and the cancer cells remaining on the top surface of the membrane were gently removed with cotton swabs. The cells migrated to the lower membrane surface were fixed with 4% paraformaldehyde for 20 min and stained with 0.2% crystal violet. All photographs were taken with a Leica inverted microscope.

#### Lung Metastasis Assay

All animal experiments and breeding comply with the guidelines for the use and feeding of Experimental Animals in Henan University. Healthy male Balb/c mice (Henan Experimental Animal Center, Zhengzhou) aged 5 weeks (weighing 18–22 g) were chosen. H22 cells (2 × 10^6^ cells/mouse) were injected *via* the tail vein for lung metastasis. To ensure the growth of pulmonary metastases in mice before drug treatment, the tumors were inoculated for 7 days. On day 8, the mice were randomly divided into three groups (n = 8 mice per group). 15f (5 mg/kg), mitoxantrone (0.04 mg/kg, positive control), or normal saline (negative control) were injected intravenously for 11 days. On day 12, the mice were anesthetized and euthanized, and their lungs were removed. After 4% paraformaldehyde fixation for 1 day, the number of pulmonary metastatic nodules of each mouse was counted. The inhibition rate was calculated. At the same time, the organs of the mice (heart, liver, kidney, lung, and spleen) were taken and weighed on the last day. The organ weight index was calculated as follows: organ index (%) = (organ weight/body weight) × 100.

### Cellular Uptake

The fluorescence intensity of 15f in cells was detected by high-content screening (HCS) (Thermo Scientific Cellomics ArrayScan VTI, Cellomics, Pittsburgh, PA, USA). Cells were seeded into 96-well plates at 5,000 cells per well for 24 h, and compounds were added. After incubation for 24 h, the fluorescence intensity was detected by HCS.

**MTT Assay**

MTT assay was used to detect the inhibition rate of cancer cells. Briefly, cells were plated in 96-well plates. After 24 h, various concentrations of compounds were subsequently added and incubated for 24 or 48 h. Then, 50 μl of MTT reagent (1 mg/ml) was added and incubated for 4 h at 37°C in a humidified incubator containing 5% CO_2_. After supernatants were removed, 100 μl of dimethyl sulfoxide was added to solubilize the crystal products at room temperature for 10 min. The absorbance was measured by a microtiter plate reader at wavelength of 570 nm. Each experiment was repeated at least three times.

### Fluorescence Intensity of Compounds in Cells

Fluorescence intensity of different concentrations of compounds (15f and HBC) in cells was detected by flow cytometry. Cells were seeded into 96-well plates at 5,000 cells per well for 24 h, and compounds (15f and HBC) (5, 10, 20, 30, 50, and 100 μM) were added, after incubation for 24 h, then cells were harvested and washed three times with PBS. Analysis was performed by flow cytometry (BD Biosciences, San Jose, CA, USA).

### Cellular Localization

The localization of 15f cells was detected by confocal microscopy (Leica, Wetzlar, German). The cells were seeded into the laser confocal cell petri dish at 5,000 cells per dish for 24 h, then 15f (5 μM) was added. After incubation for 2 h, the cells were washed three times with PBS and stained with Mito-tracker for mitochondria (Yeasen, Shanghai, China) or lysosome tracker (Jiangsu Beyotime, China) for 30 min and washed with PBS for three times. The image was captured by Leica laser confocal microscope.

### Western Blot

Cells were treated with 15f (5, 10, 15 μM) for 24 h, harvested, and centrifuged. Cell pellets were washed with cold PBS for three times and were lysed with RIPA buffer (Beyotime, China). The total concentration of protein was determined by BCA kit (Beyotime, China). The total lysates were denatured in 5×SDS-loaded buffer at 100°C. Equal amounts of total proteins were separated using 12% SDS-PAGE for 2 h and then transferred onto PVDF membrane. The membrane was sealed with 5% skimmed milk powder at room temperature in TBST for 1 h, and then incubated overnight with the corresponding primary antibody at 4°C. After washing with TBST for three times, the membrane was incubated with appropriate HRP-conjugated secondary antibody, and then washed with TBST for three times. The protein expression was detected by ECL plus reagent (Beyotime, Jiangsu, China).

## Results and Discussion

### Chemistry

**Figure 2:**
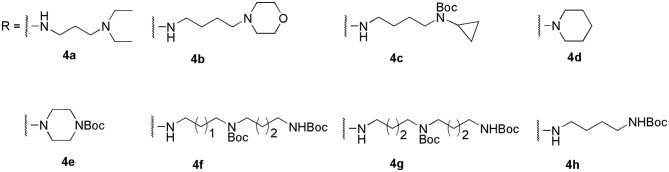
Building blocks of the designed library.

**Scheme 1:**
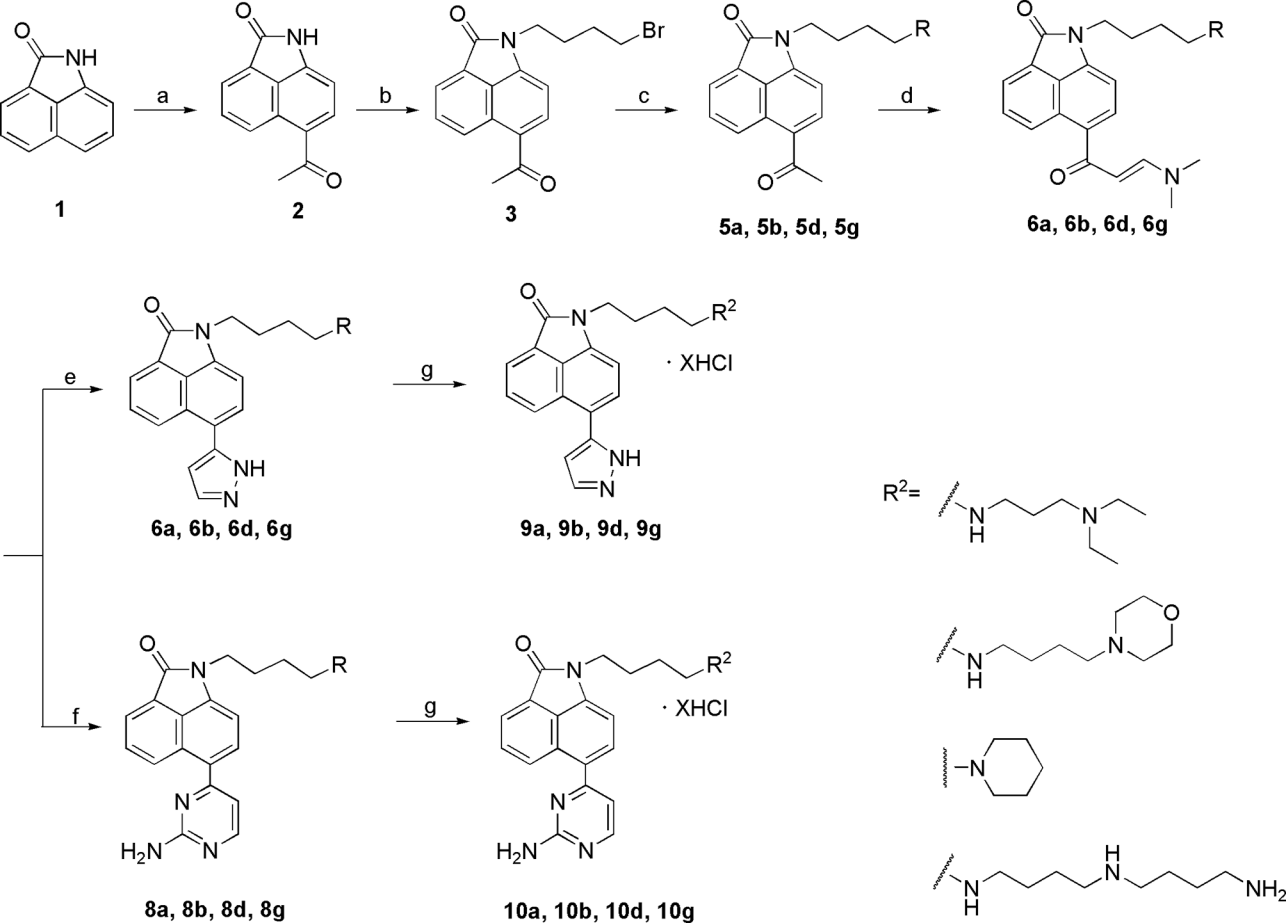
Synthesis of the target compounds 9a, 9b, 9d, 9g and 10a, 10b, 10d, 10g. Reagents and conditions: (a) acetyl chloride, CS_2_, 0°C, 1 h; (b) 1,4-butylbromide, K_2_CO_3_, CH_3_CN, reflux, 5 h; (c) i. amines(compounds 4), CH_3_CN, K_2_CO_3_, reflux, 5 h; ii. (Boc)_2_O, CH_3_OH, Et_3_N, rt, 12 h (except 5a); (d) DMF-DMA, DMF, reflux, 5 h; (e) hydrazine hydrate, EtOH, reflux, 3 h; (f’) guanidine hydrochloride, EtOH, reflux, 12 h; (g) 4 mol/L HCl, EtOH, rt, overnight.

**Scheme 2:**
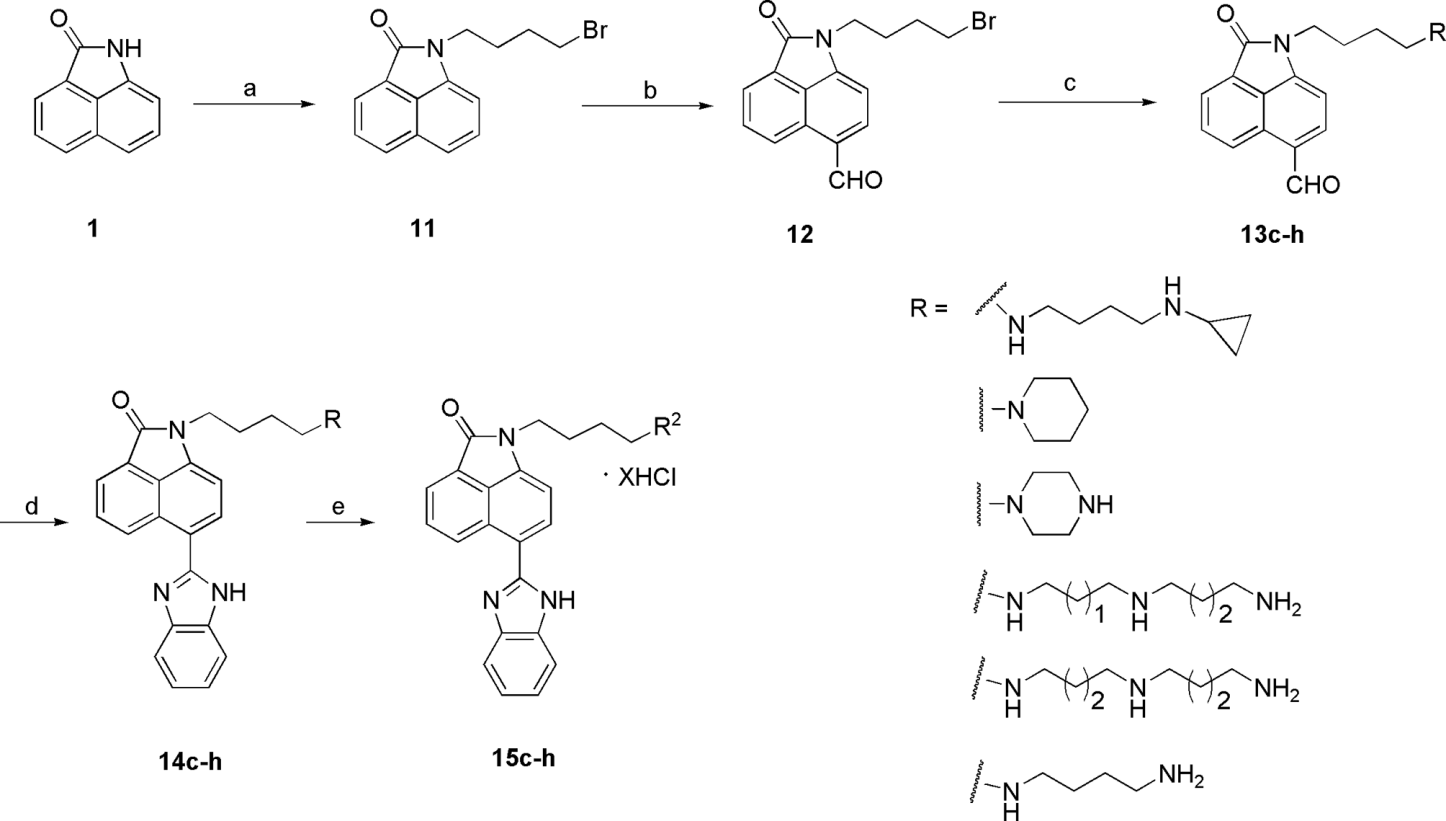
Synthesis of the target compounds 15c–h. Reagents and conditions: (a) 1,4-butylbromide, K_2_CO_3_, CH_3_CN, reflux, 5 h; (b) POCl_3_, DMF, 45°C; (c) i. amines, CH_3_CN, K_2_CO_3_, reflux, 5 h; ii. (Boc)_2_O, CH_3_OH, Et_3_N, rt, 12 h (except **13d**); (d) o-phenylenediamine, DMSO, reflux, 12 h; (e) 4 mol/L HCl, EtOH, rt, overnight.

To optimize the potency of HBC and explore its SAR, three series of derivatives were synthesized as shown in **Schemes 1**, **2**. The synthesis of compounds 9a, 9b, 9d, and 9g as well as 10a, 10b, 10d, and 10g was carried out using commercially available BIO and acetyl chloride as the starting material to yield intermediate 2 (24), which was subsequently reacted with an excess of 1,4-dibromobutane to afford intermediate 3. Next, 3 was reacted with corresponding amines/polyamines (compound 4, **Figure 2**), followed by protection with Boc_2_O (except 5d, no Boc in 5d, 6d, 7d, and 8d), which led to the formation of compounds 5a, 5b, and 5g, which were then converted into 6a, 6b, 6d, and 6g *via* reaction with DMF-DMA (25). Compounds 6a, 6b, 6d, and 6g were reacted with hydrazine hydrate or guanidine hydrochloride to afford products 7a, 7b, 7d, and 7g or 8a, 8b, 8d, and 8g, respectively (26, 27). After purification using flash column chromatography, these intermediates were stirred with 4 mol/L HCl to yield target compounds (9a, 9b, 9d, 9g, 10a, 10b, 10d, and 10g) in the form of hydrochloride salts with yields between 40 and 60%.

**Figure 3:**
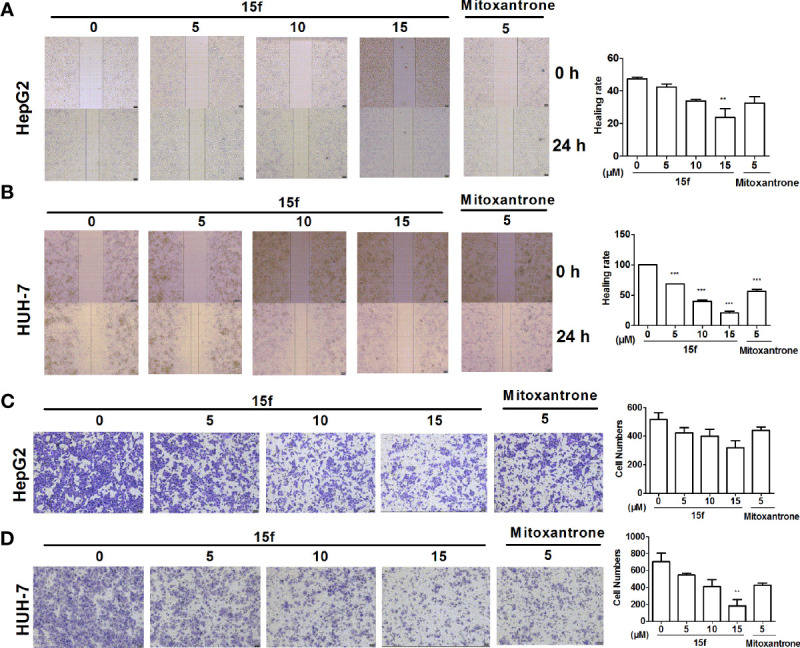
Compound 15f inhibited cell migration. **(A, B)** Effect of 15f on HepG2 and HuH-7cell migration was detected by scratch-wound assay (24 h). **(C, D)** Effect of 15f on HepG2 and HuH-7 cell migration was detected by the Transwell migration assay (without Matrigel)(24 h). *p < 0.05, **p < 0.01, and ***p < 0.001 were considered to be significant statistically.

**Figure 4:**
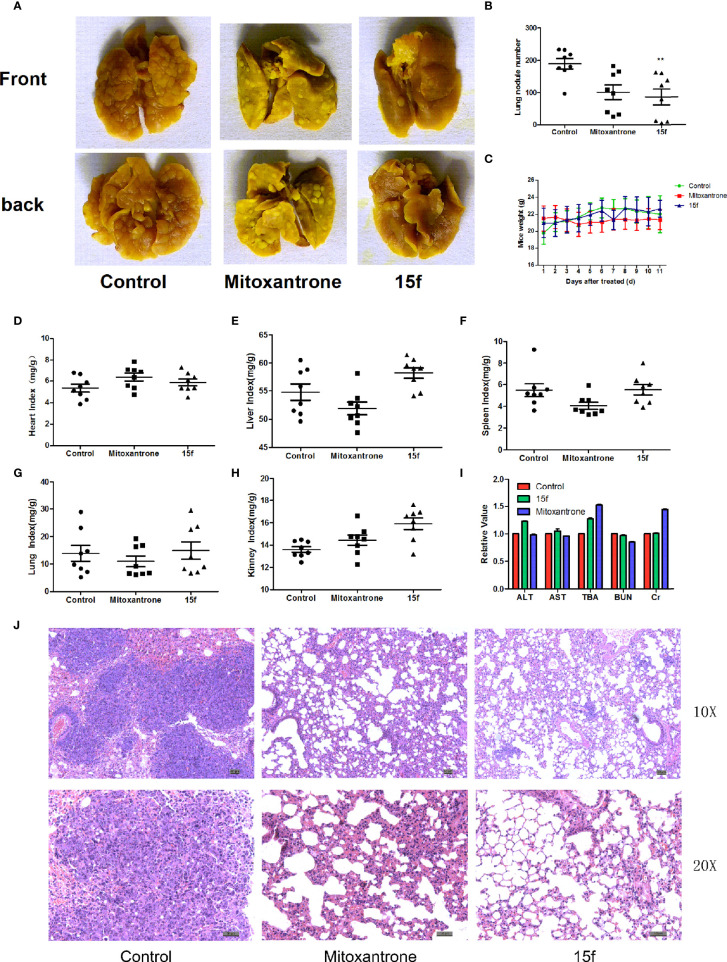
Compound 15f inhibited metastasis of liver cancer. **(A)** Metastatic lung nodules from Balb/c mice (n = 8) bearing liver cancer cells (H22) treated with mitoxantrone (0.4 mg/kg) or 15f (5 mg/kg). **(B)** Statistics of lung metastasis of hepatoma cells after treatment with mitoxantrone or 15f for 11 days. **(C)** Body weight of mice bearing hepatoma cells after treatment with mitoxantrone or 15f for 11 days. **(D–H)** Purtenance indices after treatment with mitoxantrone or 15f. **(I)** Effects of mitoxantrone or 15f on biochemical indices of liver and kidney function. **(J)** H&E staining of lung tissue. *p < 0.05, **p < 0.01, and ***p < 0.001 were considered to be significant statistically.

The synthesis of the third series of target compounds (15c–h) is shown in **Scheme 2**. Commercially available starting material 1 was converted to compound 11 and subsequently formylated using POCl_3_ to give compound 12. This was then reacted with the corresponding amines/polyamines (compound 4, **Figure 2**) and followed by protection using Boc_2_O (except 13d, no Boc in 13d and 14d) to obtain intermediates 13c, e–h. Next, compounds 13c–h were reacted with o-phenylenediamine in DMF to give the corresponding compounds, 14c–h (28). Lastly, these intermediates were reacted with 4 mol/L HCl to afford compounds 15c–h as the hydrochloride salts. The structures of all synthesized target compounds were confirmed using ^1^H NMR, ^13^C NMR, MS, and elemental analysis.

### Biological Evaluation

#### Antiproliferative Activity

The antiproliferative activity of the target compounds against four cancer cell lines was evaluated using MTT assay. The four cell lines used in our study included HCT-116 (human colon cancer), Hela (human cervical carcinoma), HepG2 (human hepatoma), and HuH-7 (human hepatoma). Mitoxantrone was used as a positive control.

As shown in **Table 1**, the two series of the synthesized target compounds (9a, 9b, 9d, 9g, and 10a–d) in which 6-H was replaced by 6-pyrazol or the aminopyrimidine group in BIO did not show improved antiproliferative activity compared to HBC or mitoxantrone. However, compounds 9g and 10g with the 4,4,4-triamine (homospermidine) motif, similar to that of HBC, were more potent than the other analogs in the two series. Similar phenomena were observed in the third subset (15c–h) of compounds that had a benzimidazole moiety. In addition, 15g and 15f, which had a triamine chain similar to that of HBC, displayed stronger inhibitory activity in comparison with other compounds and mitoxantrone (except for 15g against HCT-116). Especially, 15f was the most potent compound with IC_50_ values of 3.31 and 5.65 μM against the HCT-116 and HuH-7 cell lines, respectively, which were better than those exhibited by HBC (5.72 and 13.47 μM, respectively) and mitoxantrone (7.81 and 10.37 μM, respectively). These results indicated that linear polyamine substitution rather than branched or cyclic amino groups in the linker chain improved the antitumor activity of the synthesized compounds. The substituents on the 6-H of BIO also played an important role in antiproliferative activities. 15f showed significant antiproliferative activity against the four cancer cells; therefore, it was selected as the representative compound for subsequent experiments.

**Table 1:** *In vitro* activity of target compounds against four cancer cell lines.

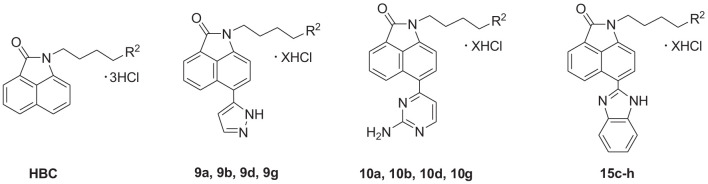						
Cpd.	R^2^	X	IC_50_^[a]^ (μM)			
			HCT-116	Hela	HepG2	HuH-7
**HBC**	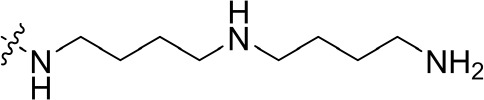	3	5.72	Nd^[b]^	5.28	13.47
**9a**	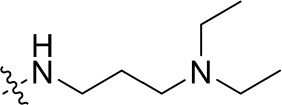	3	27.54	22.43	36.24	49.64
**9b**	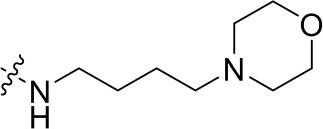	3	21.96	49.89	41.28	>50
**9d**	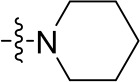	2	30.04	31.49	49.95	>50
**9g**	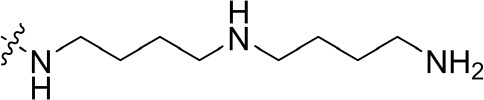	4	16.47	15.28	18.26	20.05
**10a**	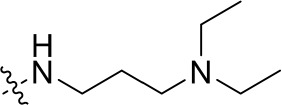	3	29.45	28.14	37.64	47.66
**10b**	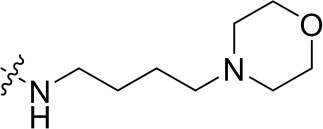	3	46.32	28.91	45.07	49.51
**10d**	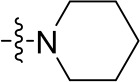	2	48.46	37.26	49.53	34.26
**10g**	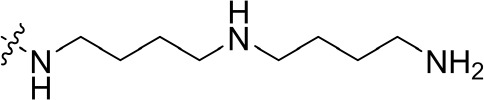	4	38.92	18.43	19.53	23.47
**15c**	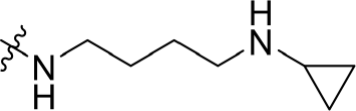	3	46.52	38.87	49.54	39.13
**15d**	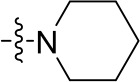	2	49.52	35.67	>50	48.93
**15e**	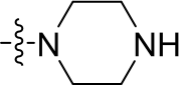	3	15.56	10.03	20.61	19.32
**15f**	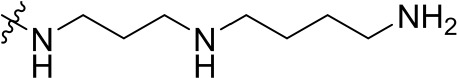	4	3.31	7.67	8.01	5.65
**15g**	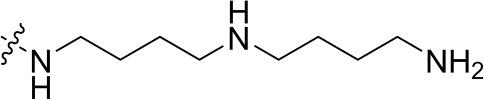	4	16.28	9.55	9.69	4.68
**15h**	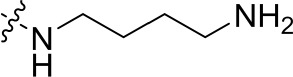	3	29.31	16.17	31.96	22.13
**Mito^[^** ^c]^	–	–	7.81	12.30	11.71	10.37

^[a]^IC_50_ represent the concentration causing 50 % growth values inhibition after treatment for 48 h. Each sample is the mean of three independent experiments; ^[b]^nd, not done; ^[c]^Mito: Mitoxantrone.

### Metastasis Inhibition *In Vitro*

Cell migration is one of the basic functions of cells that is related to cancer metastasis (29). It is also a key step in the spread of cancer from the primary masses to distant normal tissues. In order to detect whether 15f could inhibit the migration of HepG2 and HuH-7 cells, scratch-wound and Transwell migration assays were carried out. Mitoxantrone was selected as the reference compound. As illustrated in **Figures 3A–D**, our results showed that compound 15f exhibited significant inhibitory effect on the migration of cancer cells.

**Figure 5:**
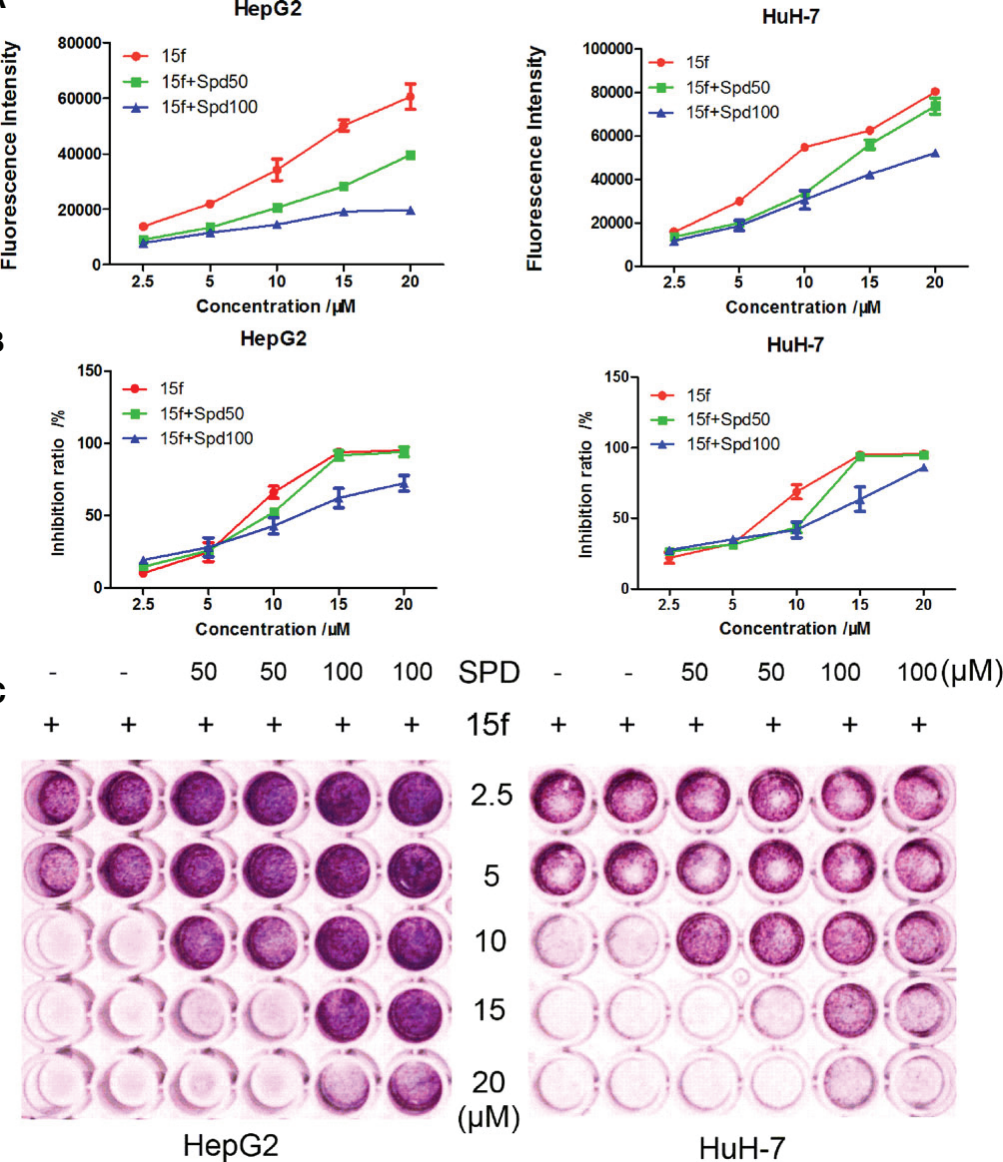
Compound **15f** affected the metabolism of polyamines in HepG2 and HuH-7 cell lines. **(A)** Spd decreased the fluorescence intensity of **15f** in cells (24 h). **(B)** Spd reversed the inhibitory effect of **15f** on cell viability (24 h). **(C)** Effect of Spd on survival of the two cell lines after treatment with **15f** (24 h).

### Metastasis Inhibition *In Vivo*

Cancer metastasis is the major factor leading to mortality in cancer patients, and the organs that are most easily affected by extrahepatic metastasis are the lungs (30). In order to evaluate the antimetastasis effect of 15f *in vivo*, a mouse model of liver cancer (pulmonary metastasis) was used. Hepatoma cells were injected into mice *via* the tail vein. After treatment with 15f (5 mg/kg), the pulmonary metastases nodules were significantly reduced (54.16%) compared to the control. Furthermore, the inhibitory effect of metastasis was found to be obviously superior compared to those observed using mitoxantrone (0.04 mg/kg, positive control, 46.37%) (**Figures 4A, B**). Meanwhile, the variations in organ weight indices implied that 15f demonstrated no obvious pathological changes in the toxicological profiles in *in vivo* experiments involving the mouse model (**Figures 4C–H**); the organ weight indices also proved that 15f was less toxic to normal tissues than mitoxantrone.

**Figure 6:**
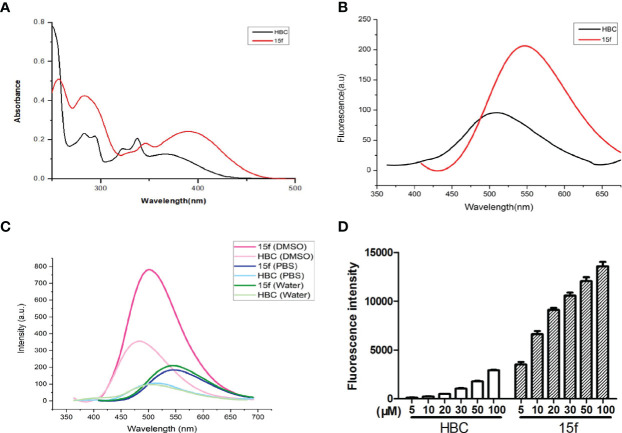
**(A)** UV absorption spectra of compound **15f** and **HBC**. Compound **15f** (50 μM in H_2_O), maximum absorption wavelength: 390 nm; Compound **HBC** (50 μM in H_2_O), maximum absorption wavelength: peak 345 nm. **(B)** Fluorescence spectra of compounds **15f** and **HBC**. Compound **15f** (25 μM in H_2_O), λ_ex_=390 nm, λ_em_=405~675 nm, slit of ExandEm were5, 5 nm, respectively; Compound **HBC** (25μM in H_2_O), λ_ex_=345 nm, λ_em_=360~675 nm, slit of ExandEm were5, 5 nm, respectively. **(C)** The fluorescence spectra of compounds **15f** and **HBC** were observed in water, PBS, and DMSO (25 μM). **(D)** Fluorescence intensity of different concentrations of compounds (**15f** and **HBC**) in HepG2 cells was detected by flow cytometry (Ex=488 nm).

In order to evaluate whether 15f had any specific effects on the functions of the liver and kidney, the blood of mice was collected according to the group, and the biochemical indices, namely, ALT (glutamic-pyruvic transaminase), AST (glutamic oxalacetic transaminase), TBA (total bile acid), BUN (urea nitrogen), and liver and kidney Cr (creatinine), were tested using an automated biochemical analyzer (Olympus, Japan) (**Figure 4I**). The biochemical indices of total bile acid and creatinine were better than those exhibited by mitoxantrone. Hematoxylin and eosin (H&E) staining further revealed that the number and size of metastatic nodules were reduced after treatment with 15f (**Figure 4J**).

Results from the *in vivo* experiments showed that 15f had a potent inhibitory effect on cancer metastasis; these results also proved the hypothesis that polyamine conjugates could serve as potential agents to prevent metastasis.

### Cellular Uptake

Compound 15f showed bright green fluorescence at maximum excitation and emission wavelengths of 390 and 550 nm, respectively. We further studied the mechanisms of uptake and distribution of 15f in cells. Natural and structurally modified polyamines are usually transported into cancer cells *via* the polyamine transporter (PAT) system (31, 32). To detect whether 15f was dependent on the PAT system to be taken up by cancer cells, spermidine, a natural PAT substrate, was selected as a representative compound and used at a concentration that did not affect cell viability or compete with potential PAT-selective polyamines. Our results showed that compared to 15f alone, 15f combined with spermidine (Spd, 50 μM, and 100 μM) decreased the fluorescence intensity in cells (**Figure 5A**). Both concentrations of Spd when combined with 15f decreased the rate of cell inhibition in hepatoma cell lines, compared to 15f alone by MTT assays (**Figure 5B**). The survival rate of cells increased in a dose-dependent manner when a combination of **15f** and Spd was used by crystal violet staining laboratory (**Figure 5C**). Therefore, 15f may have crossed the cell membrane, by virtue of being transported *via* the PAT system, and affected the metabolism of polyamines in cancer cells.

**Figure 7:**
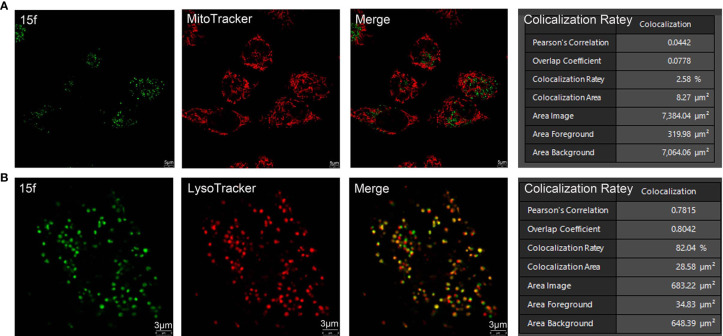
Subcellular organelle localization of **15f** in living cells. **(A)** HepG2 cells were treated with 15f (5 mM) for 2 h followed by staining with MitoTracker. **(B)** HepG2 cells were treated with 15f (5 mM) for 2 h followed by staining with LysoTracker. The images were captured on a Leica confocal microscope.

### Fluorescence Properties Study

Since compound 15f had an additional benzimidazole moiety at the 6-position, its maximum absorption and emission wavelengths (390 and 550 nm, respectively) were red-shifted compared to those of the lead compound, HBC (345 and 510 nm, respectively) (**Figures 6A, B**). In addition, results of the transient steady-state fluorescence spectrometry showed that the absolute quantum efficiency of 15f (83.53%) was much higher than that of HBC (38.45%). Therefore, it was easy to illustrate localization and demonstrate the biological imaging of 15f in living cells.

**Figure 8:**
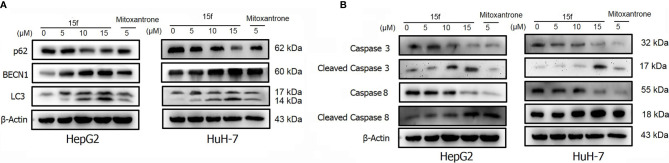
HepG2 and HuH-7 cells were treated with different concentrations of 15f for 24 h. **(A)** Effect of 15f on the expression of PPT1, CTSD, LAMP1, LAMP2, and TFEB. **(B)** Effect of 15f on the expression of E-Cadherin, N-Cadherin, and β-Catenin.

Compound 15f and HBC have strong fluorescence intensity in DMSO, compound 15f is about twice the intensity of HBC, they have weak signal in water and PBS, but the fluorescence intensity of compound 15f is still about twice the intensity of HBC (**Figure 6C**). In living HepG2 cells, the fluorescence intensity of compound 15f (5 μM) is even stronger than that of HBC (100 μM), and the difference of compound concentration is nearly 20 times (**Figure 6D**). The above results also show that the fluorescence performance of the compound has been greatly improved by structural modification. It is a step closer to the goal of integration of diagnosis and treatment.

### Localization of Sub-Organelles

The subcellular localization of 15f was detected using confocal microscopy with other organelle-specific labels (LysoTracker and MitoTracker). As shown in **Figure 7**, in 15f-treated HepG2 cells, the superposition rate with MitoTracker was only 2.58%, indicating that 15f was not likely located in mitochondria. On the other hand, the superposition rate with LysoTracker was determined to be 82.04%, which indicated that 15f was selectively located in the lysosome.

**Figure 9:**
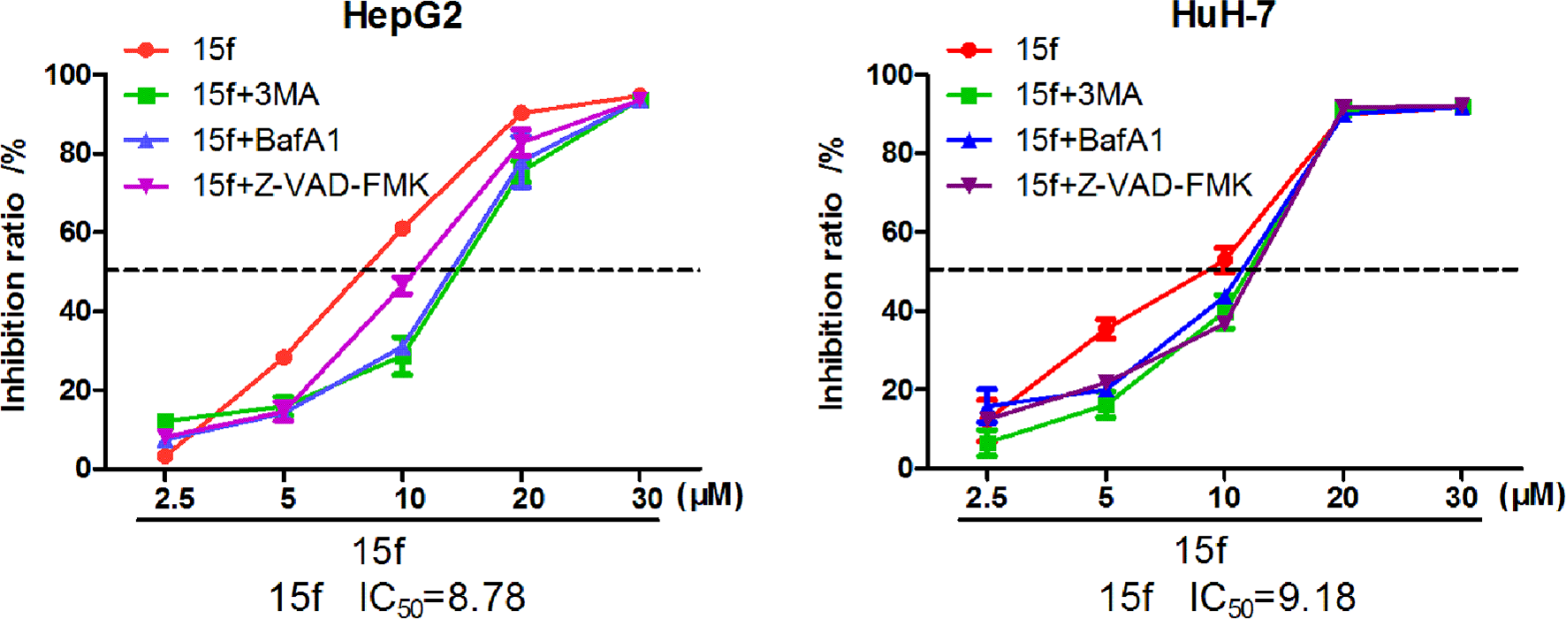
Compound 15f inhibited the growth of HepG2 and HuH-7 cells by MTT assays (24 h) combined with autophagy inhibitors (3-MA or BafA1) and apoptosis inhibitors (Z-VAD-FMK).

### Molecular Mechanism

In order to elucidate the relationship between 15f and the lysosomes, the expression of partial lysosomal-associated proteins was detected by western blot. PPT1 (palmitoyl protein thioesterase 1) is located in the lysosomes (33), and its primary function involves the catalytic reactions of depalmitoylation of palmitoylated proteins. In other words, it intervenes in several biological processes of the cell by regulating the dynamic modification of the palmitoylation process (palmitoylation- depalmitoylation) in specific cysteine residues of proteins. It was reported by Rebecca et al. that PPT1 could represent a new target in the management of cancer, and dimeric chloroquine (DC661) and dimeric quinacrines (DQ661) could exert antitumor effect by inhibiting the expression of PPT1, reducing acidity of lysosomes, and inhibiting autophagy (11, 12). However, our results showed that compound 15f increased the expression of PPT1 and enhanced autophagy (**Figure 8**). This unexpected result sparked our interest to further explore its activity.

**Figure 10:**
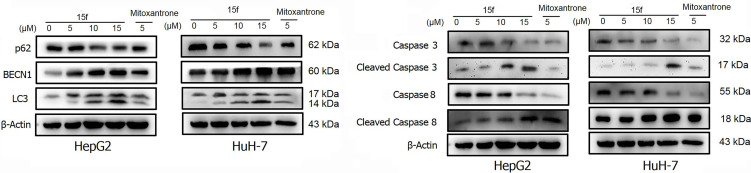
HepG2 and HuH-7 cells were treated with 15f or inhibitors for 24 h. **(A)** Effect of 15f on the expression of p62, BECN1, and LC3-II. **(B)** Effect of 15f on the expression of caspase 3, cleaved caspase 3, caspase 8, and cleaved caspase 8.

Cathepsins are overexpressed in many tumor cells and mainly in the lysosomes. Intracellular cathepsins promote metastasis by activating associated proteases and degrading proteins of the extracellular matrix. Treatment with 15f led to the upregulation of cathepsin D (CTSD) in HepG2 and HuH-7 cells in a dose-dependent manner (**Figure 8A**). It has been reported that the overexpression of CTSD enhances cancer invasion and metastasis in various malignant cancers (34). This could be one of the reasons why the activity of 15f in inhibiting tumor migration was lower than that of HBC (downregulated CTSD).

LAMP1 and LAMP2 are lysosomal-associated transmembrane proteins, which serve as lysosomal markers and play important roles in lysosome-mediated physiological processes including participation in autophagy. As shown in **Figure 8A**, the downregulation of LAMP1 and upregulation of LAMP2 in two cell lines in a dose-dependent manner indicates that lysosomal function is altered by 15f.

TFEB (Transcription Factor EB) plays an important role in ontogeny and development. The biological functions of TFEB include roles in angiogenesis, renal cell carcinoma, lysosome synthesis, and autophagy. TFEB is closely related to the functions of lysosomes. Results showed that TFEB was downregulated after treatment with 15f, thereby confirming the effect of this compound on lysosomes.

Upregulated expression of TFEB is associated with the migration of cancer cells. E-Cadherin, N-Cadherin, and β-Catenin are key proteases in migration and play crucial roles in promoting tumor-cell migration. To detect the effect of 15f on migration, the expression of E-Cadherin, N-Cadherin, and β-Catenin was measured using western blot assays. Results of the western blot analysis revealed that 15f upregulated the expression of E-cadherin and downregulated the expression of N-cadherin and β-catenin in the two cell lines in a dose-dependent manner (**Figure 8B**), which indicated that the migration of cancer cells was inhibited.

### 15f-Induced Autophagy and Apoptosis

Autophagy is a complex catabolic process in which cellular components are transported to lysosomes for degradation. The localization of 15f in lysosomes suggested the crucial role of the organelle in 15f-induced autophagy. 3-methyladenine (3-MA) (21) is an inhibitor of PI3K. It is widely used as an inhibitor of autophagy by inhibiting class III PI3K. Bafilomycin A1 (BafA1) is a specific inhibitor of vesicle H+ ATPase (V-ATPase). It blocks the fusion of autophagy and lysosome and inhibits late autophagy. It is also commonly used as an autophagy inhibitor. Therefore, 3MA and BafA1 were used to determine the accuracy of our hypothesis. As shown in **Figure 9**, addition of 3-MA and BafA1 protected cells from the growth-inhibition effect by 15f. The effect of inversion is particularly significant at 10 μM. These preliminarily results indicated that 15f induced autophagy.

**Figure 11:**
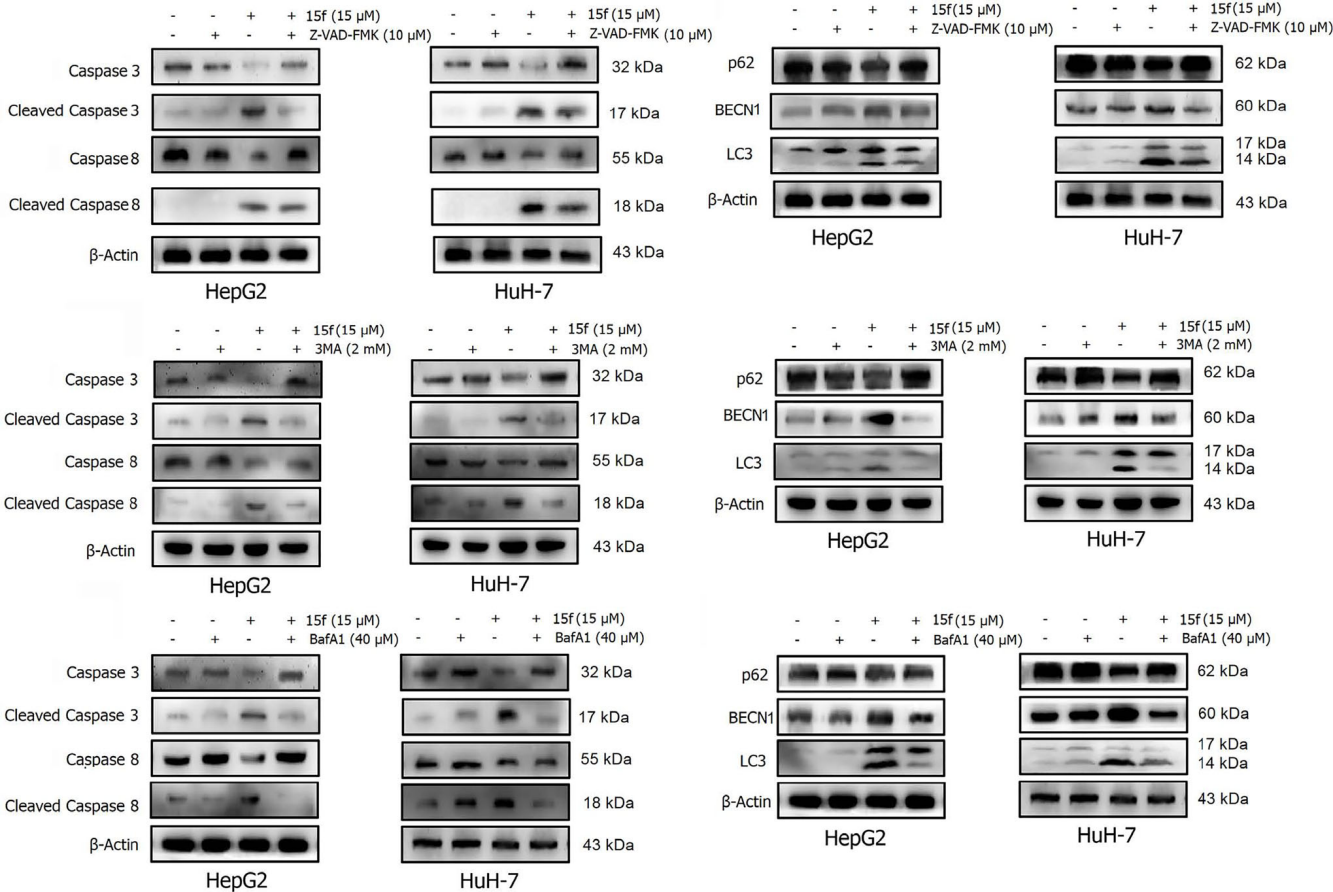
Effects of **15f** (24 h) on the expression of apoptotic proteins and autophagy proteins. Expression of these proteins was reversed in HepG2 and HuH-7 cells after addition of **(A, B)** Z-VAD-FMK (10 μM), **(C, D)** 3-MA (2 mM), and **(E, F)** BafA1 (40 μM).

Apoptosis or programmed cell death is a carefully controlled, energy-dependent process. Z-VAD-FMK is one of the inhibitors of apoptosis (21). It was used to determine whether 15f induced apoptosis. As shown in **Figure 9**, the addition of Z-VAD-FMK protected cells from the growth-inhibition effect of 15f. The effect of inversion is particularly significant at 5 and 10 μM. These results indicated that 15f induced apoptosis.

To further verify whether 15f induced autophagy and apoptosis simultaneously, autophagy- and apoptosis-associated proteins were quantified (**Figure 10A**). Results showed that 15f downregulated p62 and upregulated BECN1 and LC3. Since p62, BECN1, and LC3 are key proteases involved in autophagy, treatment with 15f induced autophagy in the two cell lines. As shown in **Figure 10B**, 15f upregulated the levels of cleaved caspase 3 and cleaved caspase 8 and downregulated those of caspase 3 and caspase 8. Since caspases 3 and 8 are implicated as key mediators of apoptosis, it could be inferred that 15f induced apoptosis in HepG2 and HuH-7 cells. These findings collectively indicated that 15f induced both autophagy and apoptosis.

### Relationship Between Autophagy and Apoptosis

In order to determine the relationship between apoptosis and autophagy induced by 15f, additional studies were conducted. After addition of the apoptosis inhibitor, Z-VAD-FMK, the expressions of apoptotic proteins Caspase 3, Cleaved Caspase 3, and Caspase 8 were found to be reversed obviously in HepG2 and HuH-7 cells, and Cleaved Caspase 8 was reversed more evidently in HuH-7 than in HepG2 cells. These results demonstrated the occurrence of apoptosis at the protein level. Concurrently, the expression of autophagy proteins such as p62 and BECN1 was also reversed and LC3 was reversed more obviously in HepG2 than in HuH-7 cells. To a certain extent, the results indicate that apoptosis may promote autophagy (**Figures 11A, B**).

A similar phenomenon occurred after the addition of 3-MA, in which both autophagy- and apoptosis-associated proteins were reversed (**Figures 11C, D**). These results indicated a relationship between apoptosis and autophagy in 15f-treated cancer cells in a loop. Bafilomycin A1 (BafA1) is a specific inhibitor of the vesicular H+−ATP enzyme (V-ATPase); it blocks the fusion of autophagosomes and lysosome and inhibits late autophagy; therefore, it is often used as an inhibitor of autophagy and lysosomal degradation (35–37). Our results showed that the expression of apoptosis and autophagy proteins was also reversed after treatment of HepG2 and HuH-7 cells with BafA1 (**Figures 11E, F**).

Results of western blot (**Figure 11**) indicated that **15f** was involved in the induction of apoptosis and autophagy; these two processes mutually promoted each other. These results were consistent with those obtained from the MTT assay (**Figure 9**).

## Conclusion

In this study, using HBC as the lead compound, we synthesized three series of compounds and determined their activities *in vitro*. Our results showed that 15f had potent inhibitory effect on cancer migration both *in vitro* and *in vivo*. The uptake of 15f by cancer cells partially depended on the PAT system. Spd and other compounds affected the entry of 15f into cancer cells and ultimately altered the efficacy of 15f. Upon entering the cells, a significant amount of 15f was distributed in the lysosome, which consequently caused cell autophagy and apoptosis. Through MTT assays and western blot experiments, it can be proved that there may be a mutually promoting relationship between autophagy and apoptosis, eventually leading to the death of cancer cells together. Besides, 15f exhibited a stronger green fluorescence than the lead compound, HBC, implying that it can be used as a novel fluorescence probe to indicate the location of lysosomes. To summarize, compound **15f** was determined to be a valuable dual-functional agent and can be further developed as a promising lead compound in the management of metastatic liver cancer and a diagnostic tool in bio-imaging.

## Data Analysis

All the data are presented as the mean ± SD and analyzed using Student’s t test or analysis of variance (ANOVA) followed by q-test. *p < 0.05, **p < 0.01, and ***p < 0.001 were considered to be significant statistically.

## Data Availability Statement

The original contributions presented in the study are included in the article/**Supplementary Material**. Further inquiries can be directed to the corresponding authors.

## Ethics Statement

The animal study was reviewed and approved by the Biomedical Research Ethics Committee, Henan University.

## Author Contributions

JL, CW, SX, and YW conceived and designed the study. JL, SC, YZ, HG, TW, XG, and CZ performed the experiments and analyzed the data. WL, LC, and FD conducted data curation and analyzed the data. JL, WL, and YW were responsible for software analysis and data visualization. CW, WL, and YW reviewed and edited the manuscript. All authors contributed to the article and approved the submitted version.

## Funding

This work was supported by the National Natural Science Foundation of China (No. U1704176), China Postdoctoral Science Foundation (2020M682374), Post doctoral Research Sponsorship of Henan Province (2015035), Program of First-Class Discipline Cultivation Project of Henan University (CJ1205A0240010), Kaifeng Social Development Science and Technology Project (1503010), Program for Innovative Research Team (Science and Technology in University of Henan Province) (No. 19IRTSTHN004), and the Young Talents Project of Medical School of Henan University (No. 2019005).

## Conflict of Interest

The authors declare that the research was conducted in the absence of any commercial or financial relationships that could be construed as a potential conflict of interest.

## Publisher’s Note

All claims expressed in this article are solely those of the authors and do not necessarily represent those of their affiliated organizations, or those of the publisher, the editors and the reviewers. Any product that may be evaluated in this article, or claim that may be made by its manufacturer, is not guaranteed or endorsed by the publisher.

## Abbreviations

HCC, Hepatocellular carcinoma; CQ, chloroquine; BIO, Benzo[cd]indol-2(1H)-one; HCT-116, human colon cancer; Hela, human cervical carcinoma; HepG2, human hepatoma; HuH-7, human hepatoma; Mito, Mitoxantrone; ALT, glutamic-pyruvic transaminase; AST, glutamic oxalacetic transaminase; TBA, total bile acid; BUN, urea nitrogen; H&E, Hematoxylin and eosin; PPT1, palmitoyl protein thioesterase 1; DC661, dimeric chloroquine; DQ661, dimeric quinacrines; CTSD, cathepsin D; TFEB, Transcription Factor EB; 3-MA, 3-methyladenine; BafA1, Bafilomycin A1; V-ATPase, Vacuolar-type ATPase; Cr, creatinine; SAR, structure-activity relationship; HBC, 1-{4-[4-(4-Aminobutylamino)butylamino]butyl]}-benzo[*cd*]indol-2(1H)-one trihydrochloride (homospermine-benzo[cd]indol-2(1H)-one conjugate).
