# Supplementary material for: Design, Synthesis, and Biological Evaluation of Benzo[cd]indol-2(1H)-ones Derivatives as a Lysosome-Targeted Anti-metastatic Agent
Source: Front Oncol. 2021 Aug 27;11:733589. doi: 10.3389/fonc.2021.733589 (PMC8446683; doi:10.3389/fonc.2021.733589)
Supplement: Supplementary file 1 [file DataSheet_1.docx]

**Supporting Information**

Design, synthesis and biological evaluation of benzo[cd]indol-2(1H)-ones derivatives as a lysosome-targeted anti-metastatic agents

Jinghua Li^1^^†^, Shuai Chen^1†^, Yancong Zhao^2^, Huiyuan Gong^1^, Tong Wang^3^, Xiaoling Ge^4^, Yuxia Wang^4*^, Chenguang Zhu^1^, Liang Chen^1^, Fujun Dai^1^, Songqiang Xie^3^, Chaojie Wang^1^, Wen Luo^1*^

^1^Jinghua Li^†^, Shuai Chen^†^, Huiyuan Gong, Chenguang Zhu, Liang Chen, Fujun Dai, Chaojie Wang, Wen Luo^*^

^1^Key Laboratory of Natural Medicine and Immuno-Engineering, Henan University, Kaifeng 475004, China

^2^Yancong Zhao

^2^The First Affiliated Hospital,Henan University, Kaifeng 475004, China

^3^Songqiang Xie, Tong Wang

^3^Institute of Chemical Biology, School of Pharmacy, Henan University, Kaifeng 475004, China

^4^Xiaoling Ge, Yuxia Wang^*^

^4^College of Chemistry and Chemical Engineering Henan University, Kaifeng 475004, China

**^*^Correspondence:**

Yuxia Wang, Email: wangyuxia@henu.edu.cn

Wen Luo, Email: luowen83@henu.edu.cn

^†^ These authors contributed equally to this work.

**Table of Contents**

| **Supplementary Figures** | **Page** |
| --- | --- |
| Figure S1. NMRand MS of target compounds | S2-S29 |

**Figure S1** NMR spectrum and MSof targets

{1-[4-(3-diethylaminopropyl) aminobutyl]}-[6-(1H-pyrazol-5-yl)] benzo[cd]indol-2(1H)-one trihydrochloride (**9a**)


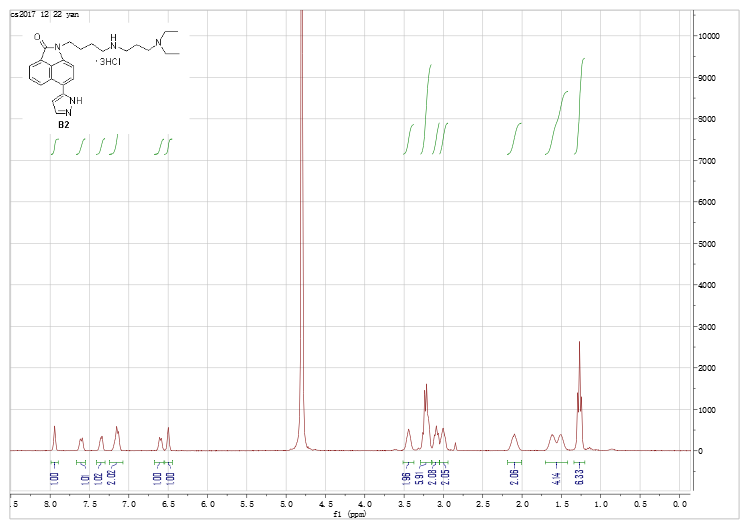


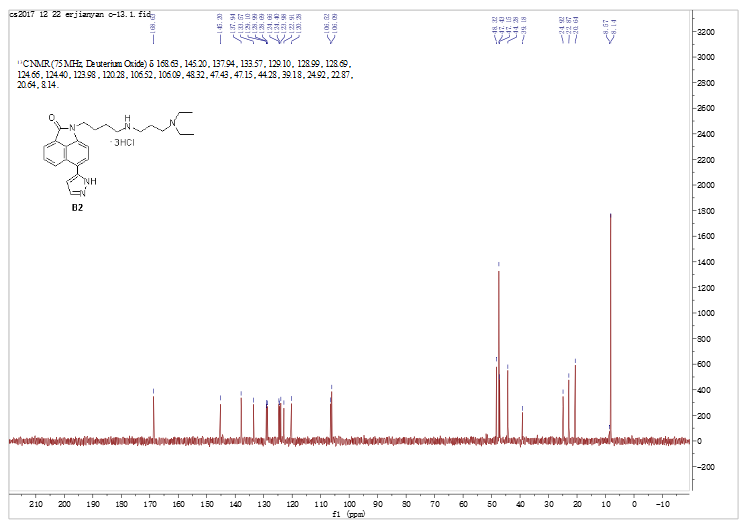


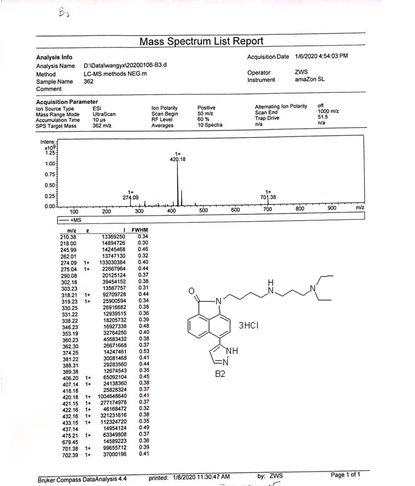


{1-[4-(4-morpholinobutyl)aminobutyl]}-[6-(1H-pyrazol-5-yl)]benzo[cd]indol-2(1H)-one trihydrochloride (**9b**)

**
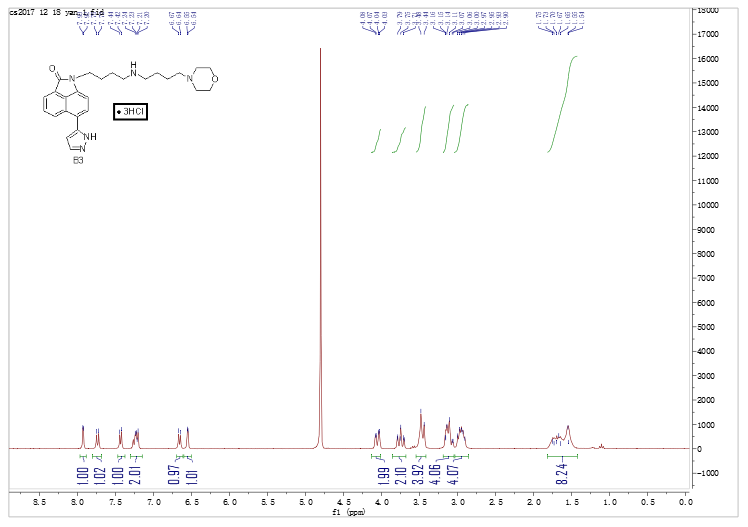
**

**
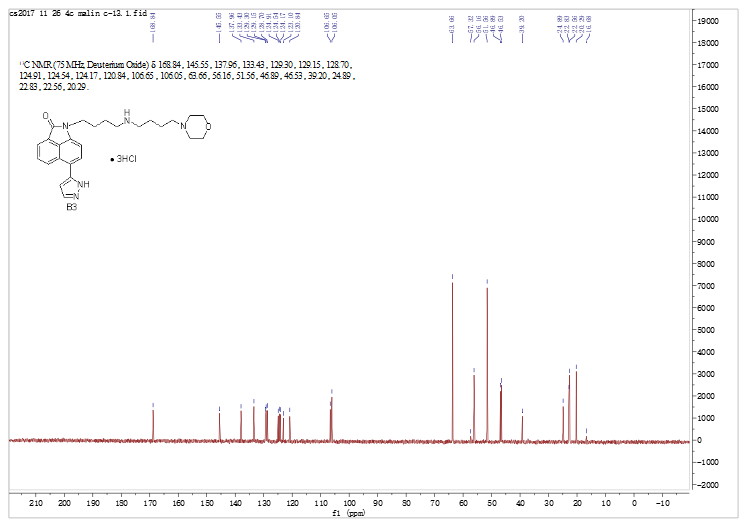
**

**
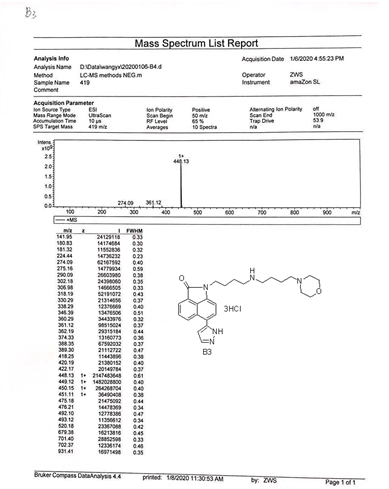
**

(1-(4-(1-piperidyl)butyl)-[6-(1H-pyrazol-5-yl)]benzo[cd]indol-2(1H)-one dihydrochloride (**9d**)


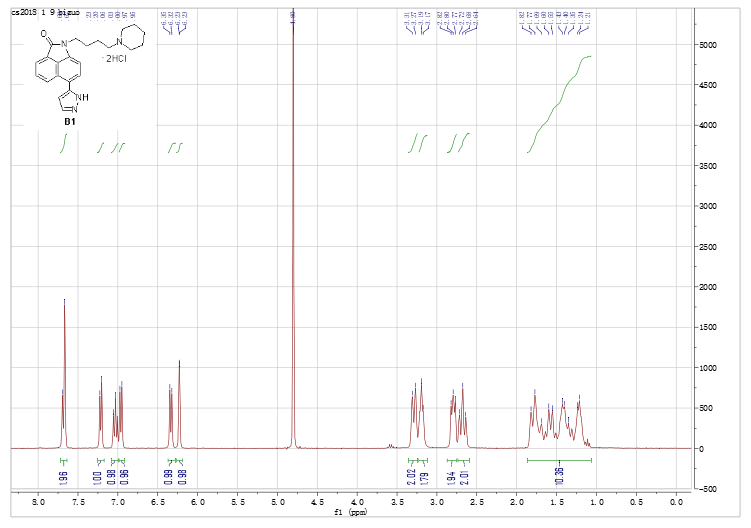


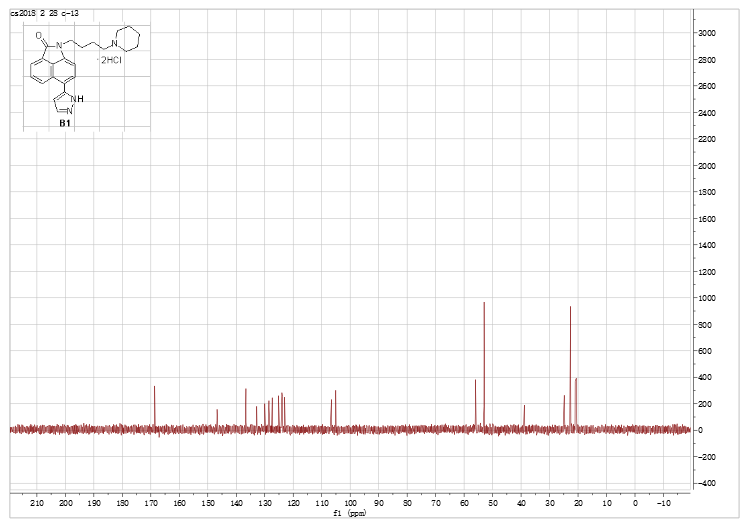


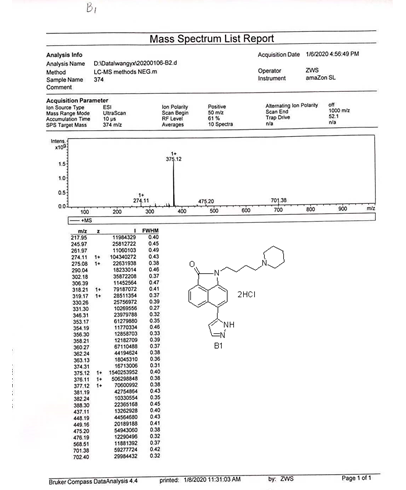


{1-[4-4-(4-aminobutyl) aminobutyl aminobutyl]}-[6-(1H-pyrazol-5-yl)]benzo[cd]indol-2(1H)-one tetrahydrochloride (**9g**)

**
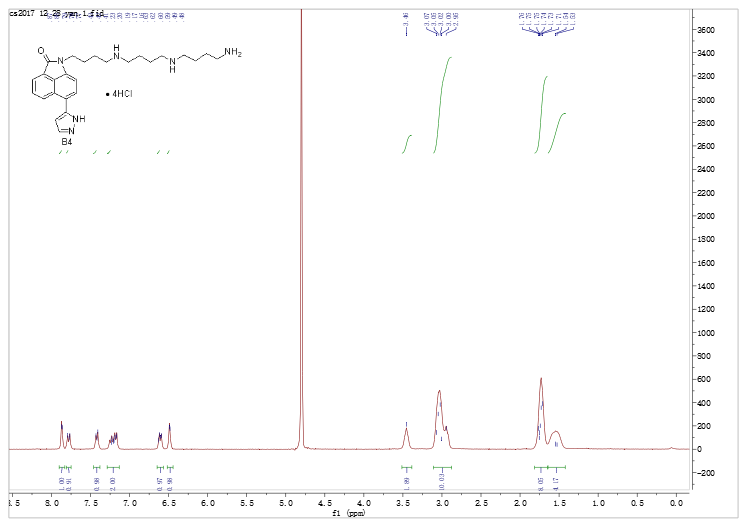
**

**
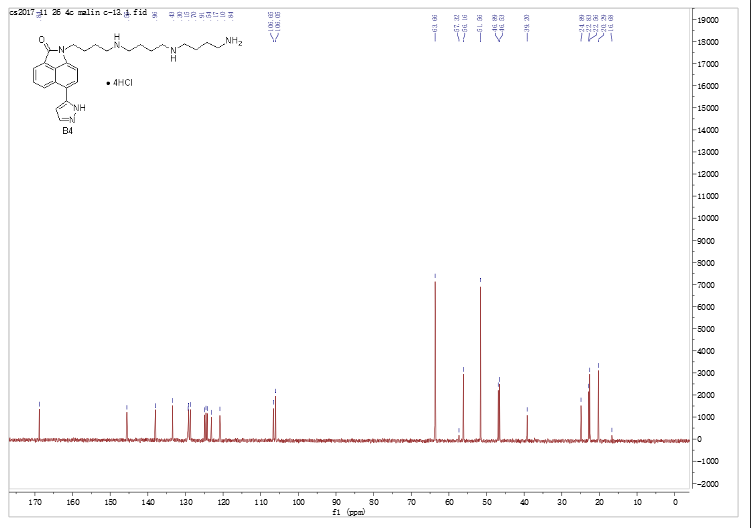
**

**
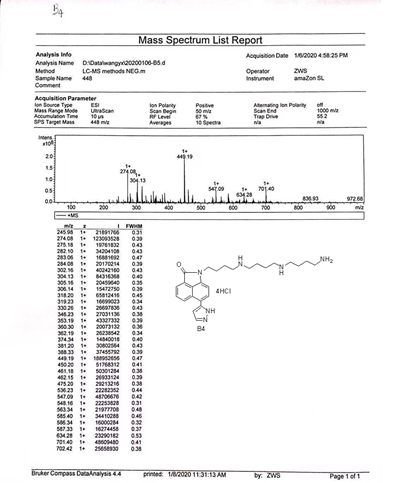
**

{1-[4-(3-diethylaminopropyl) aminobutyl]}-[6-(2-aminopyrimidin-4-yl)] benzo[cd]indol-2(1H)-one trihydrochloride (**10a**)

**
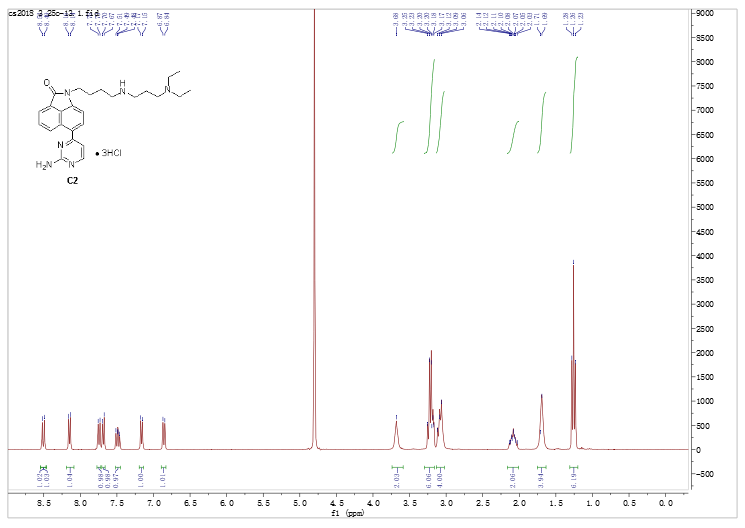
**

**
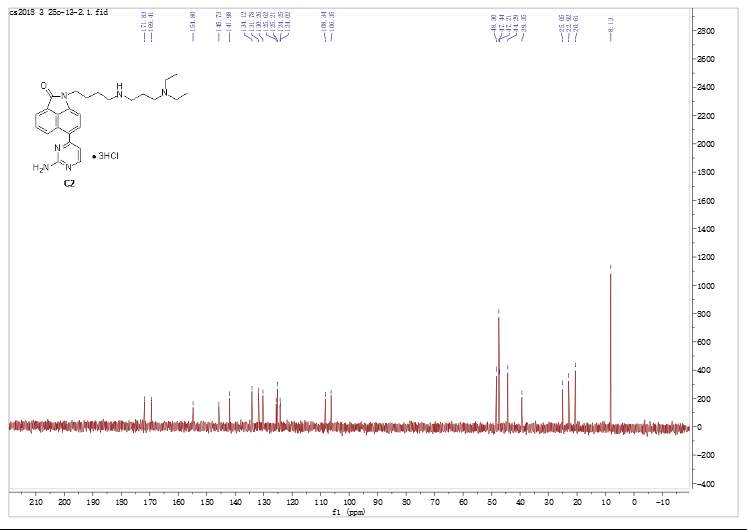
**

**
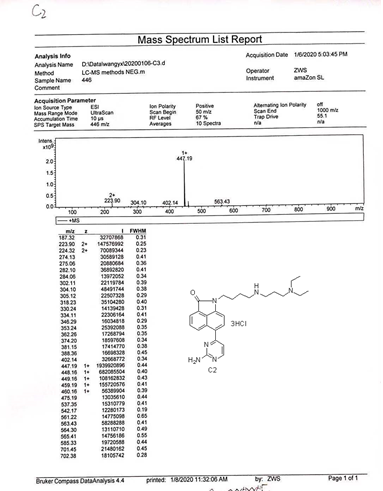
**

{1-[4-(4-morpholinobutyl)aminobutyl]}-[6-(2-aminopyrimidin-4-yl))]benzo[cd]indol-2(1H)-one trihydrochloride (**10b**)

**
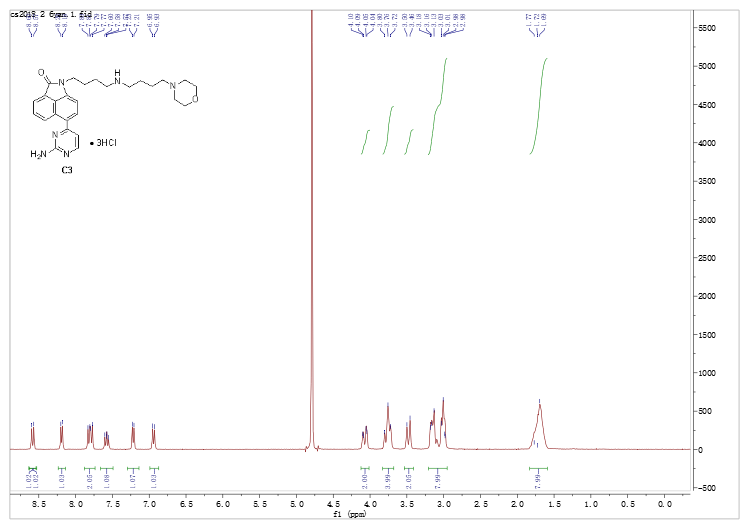
**

**
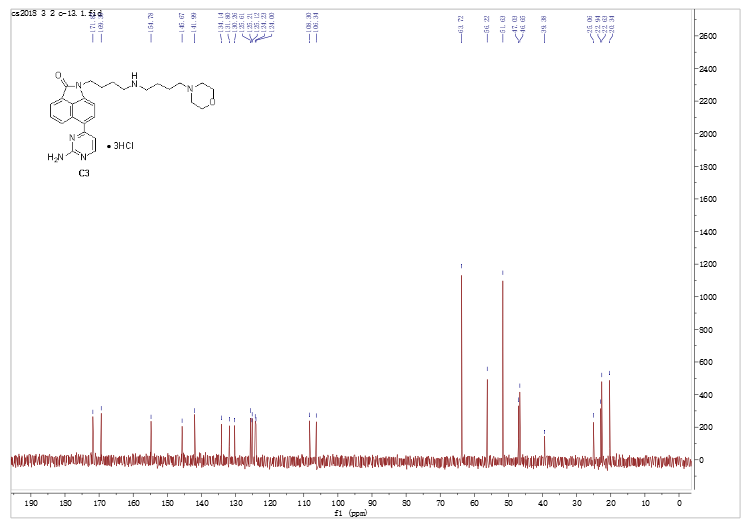
**


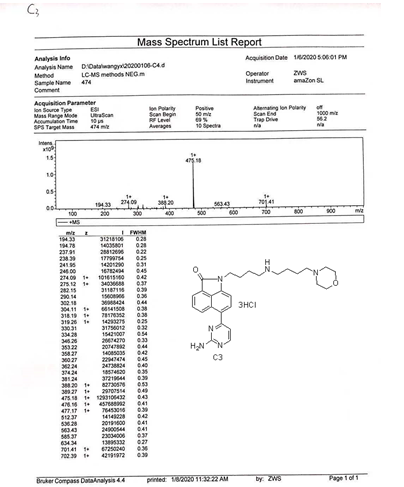


{1-[4-(1-piperidyl)butyl]}-[ 6-(2-aminopyrimidin-4-yl)]benzo[cd]indol-2(1H)-one dihydrochloride (**10d**).

**
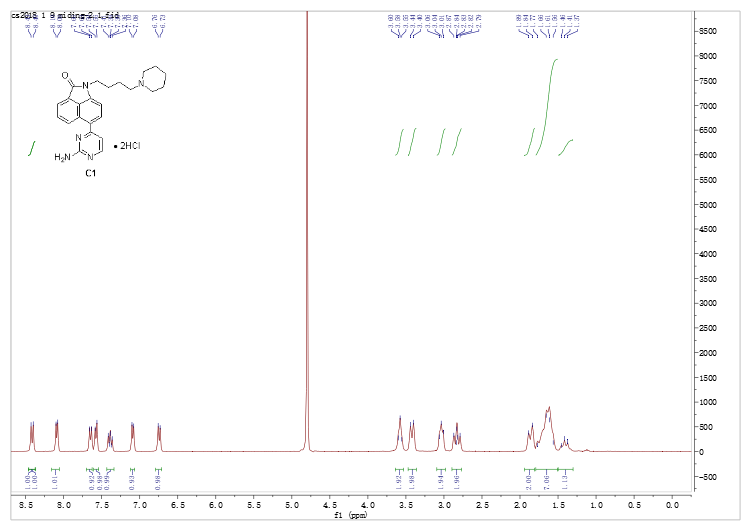
**

**
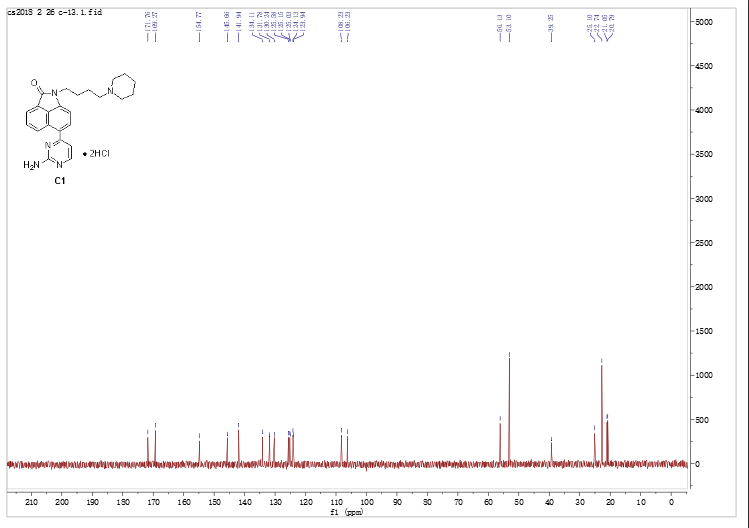
**

**
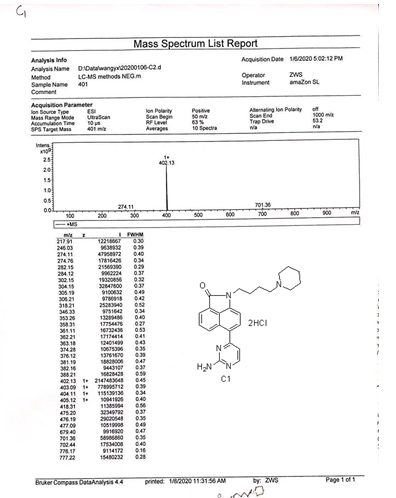
**

{1-[4-(4-aminobutyl)aminobutylaminobutyl]}-[6-(2-amino-pyrimidin-4-yl)]benzo[cd]indol-2(1H)-one tetrahydrochloride (**10g**)

**
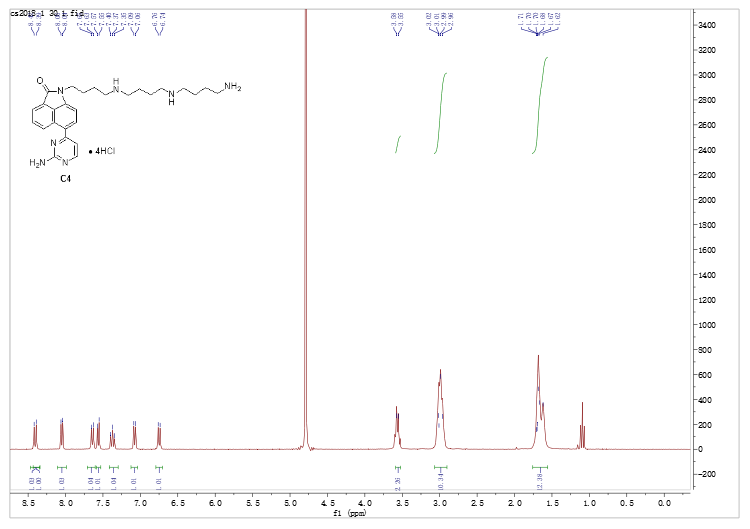
**

**
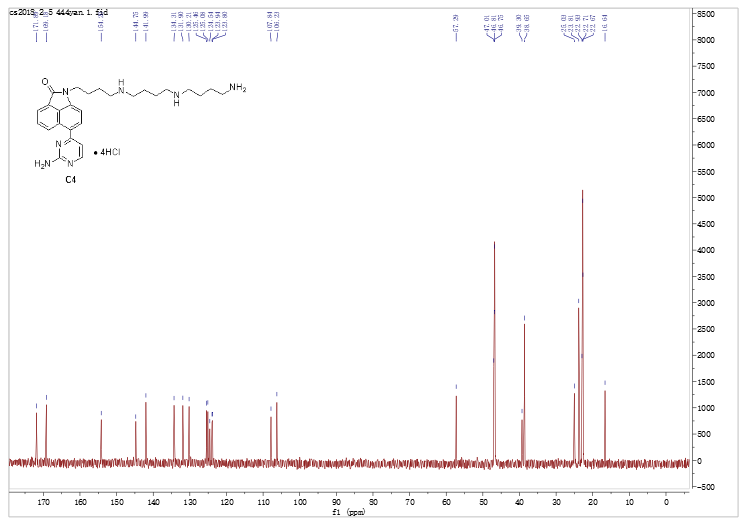
**


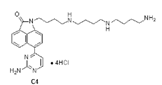


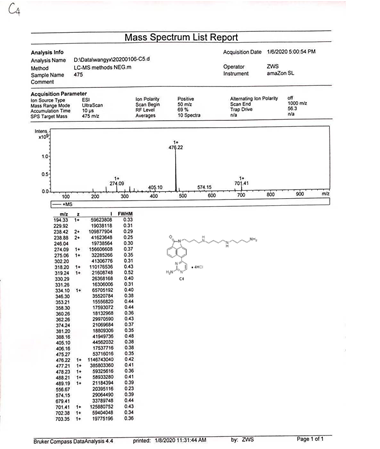


{1-[4-(4-cyclopropylamino)aminobutyl]}-[6-(1H-benzo[d]imidazol-2-yl)]benzo[cd]indol-2(1H)-one trihydrochloride (**15c**)

**
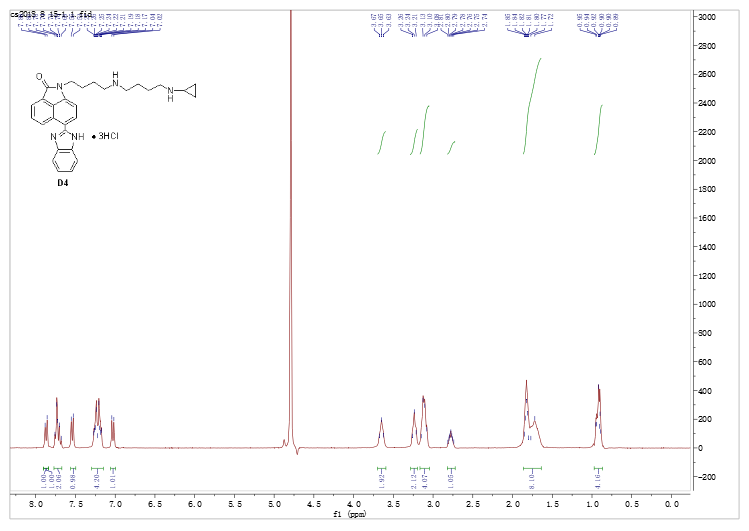
**

**
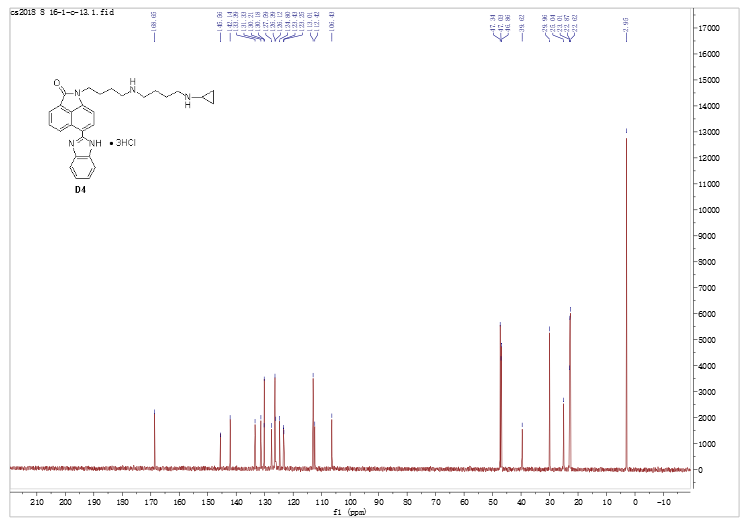
**

**
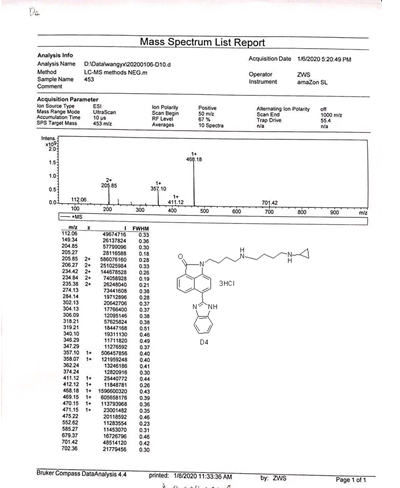
**

{1-[4-(1-piperidyl)butyl]}-[6-(1H-benzo[d]imidazol-2-yl)]benzo[cd]indol-2(1H)-one (**15d**)


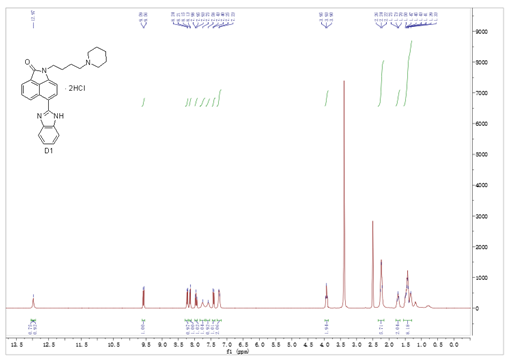


**
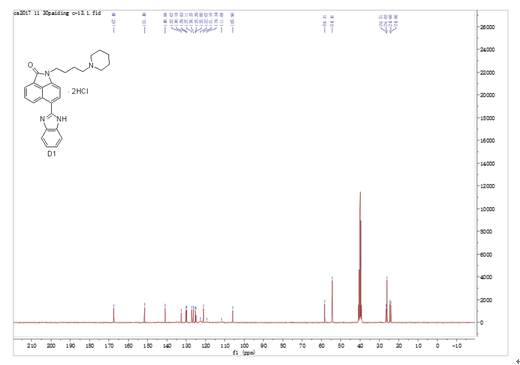
**

**
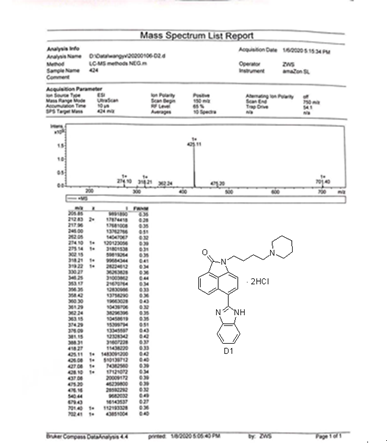
**

{1-[4-(1-piperazinyl)butyl]}-[6-(1H-benzo[d]imidazol-2-yl)]benzo[cd]indol-2(1H)-one trihydrochloride (**15e**)

**
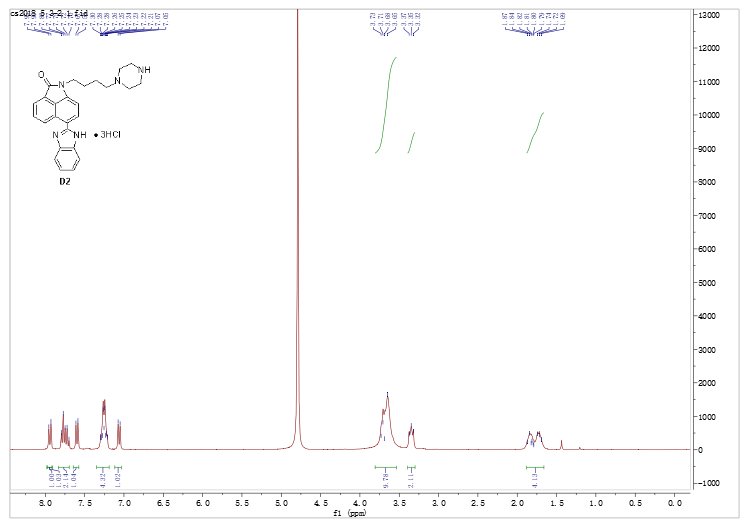
**

**
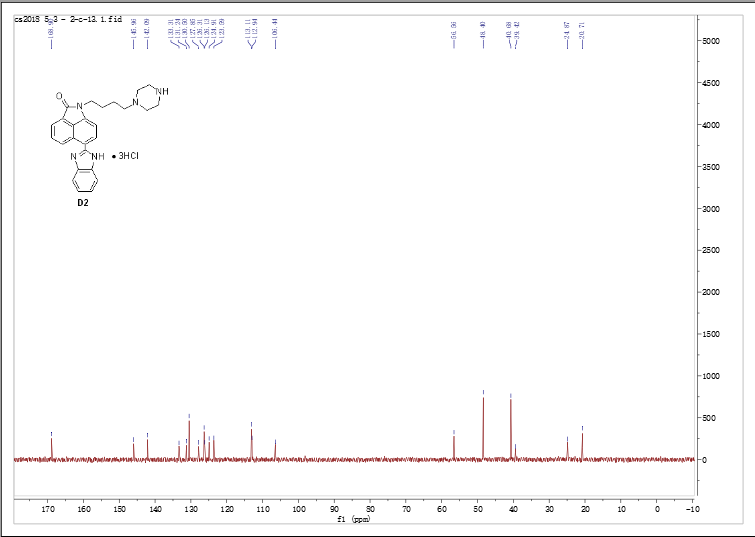
**

**
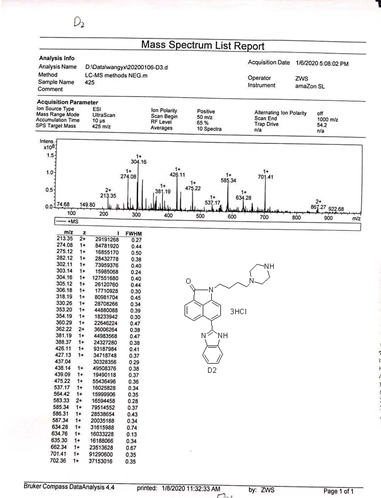
**

{1-[4-(3-(4-aminobutyl)aminopropyl)aminobutyl]}-[6-(1H-benzo[d]imidazol-2-yl)]benzo[cd]

indol-2(1H)-one tetrahydrochloride (**15f**)

**
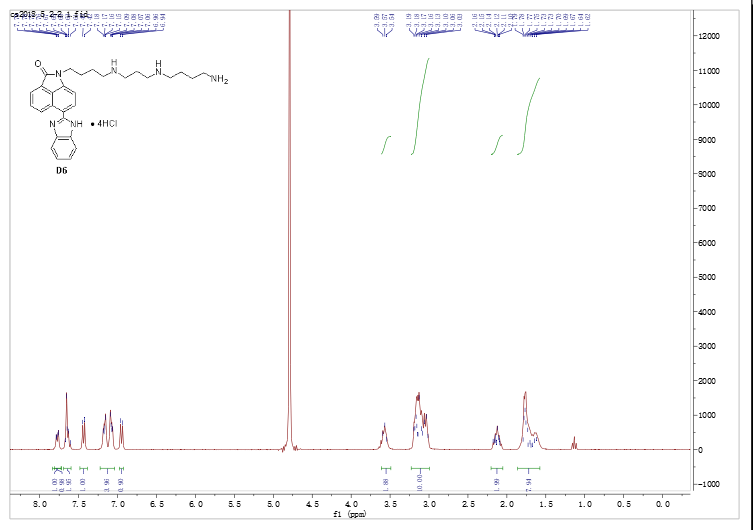
**

**
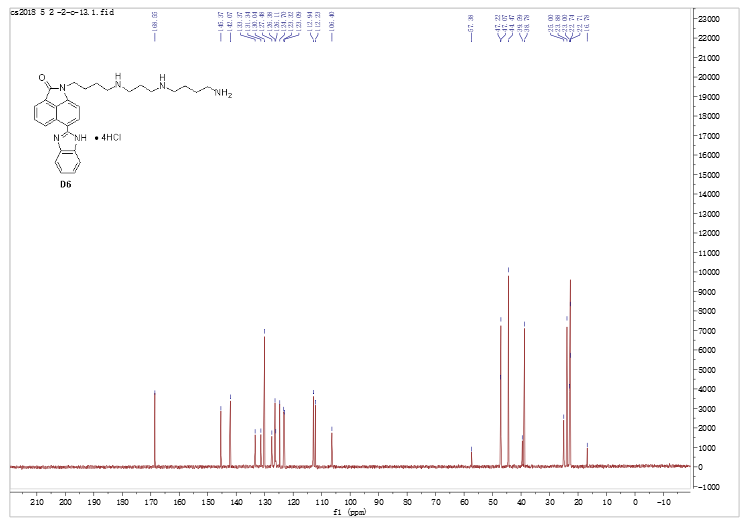
**

**
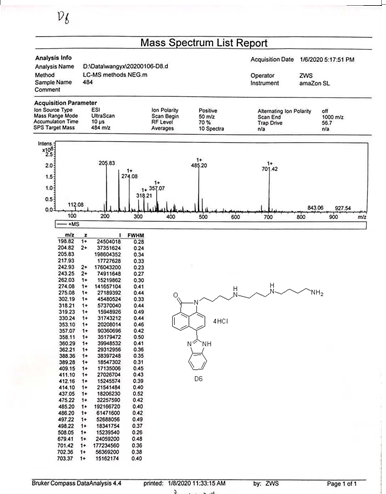
**

{1-[4-(4-(4-aminobutyl)aminobutyl)aminobutyl]}-[6-(1H-benzo[d]imidazol-2-yl)]benzo[cd]indol-2(1H)-one tetrahydrochloride (**15g**)

**
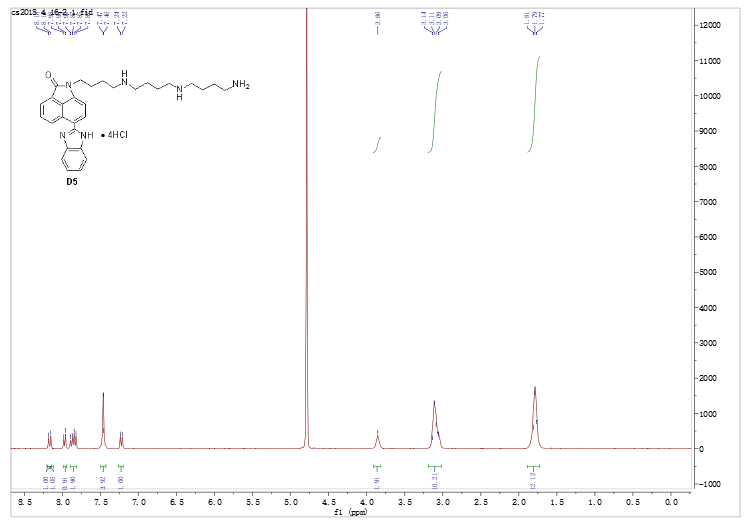
**

**
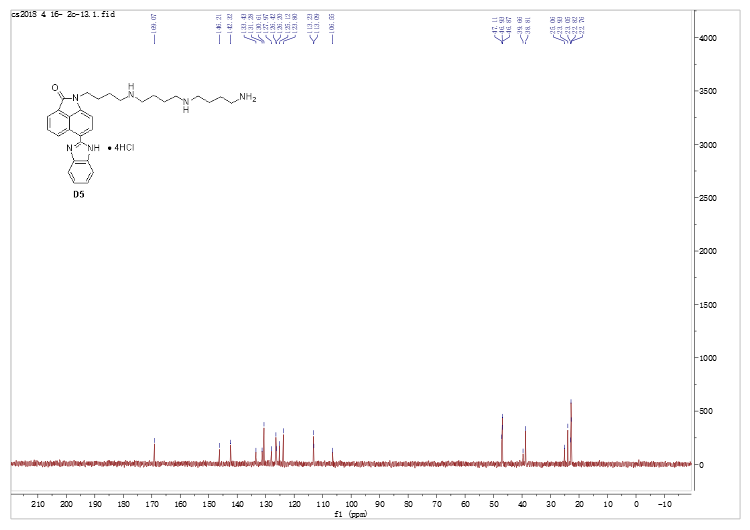
**

**
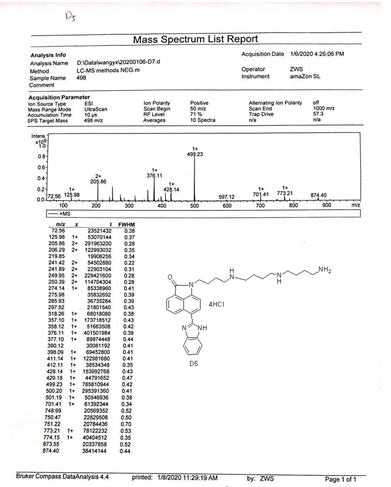
**

{1-[4-(4-aminobutyl)aminobutyl]}-[6-(1H-benzo[d]imidazol-2-yl)]benzo[cd]indol-2(1H)-one trihydrochloride (**15h**)

**
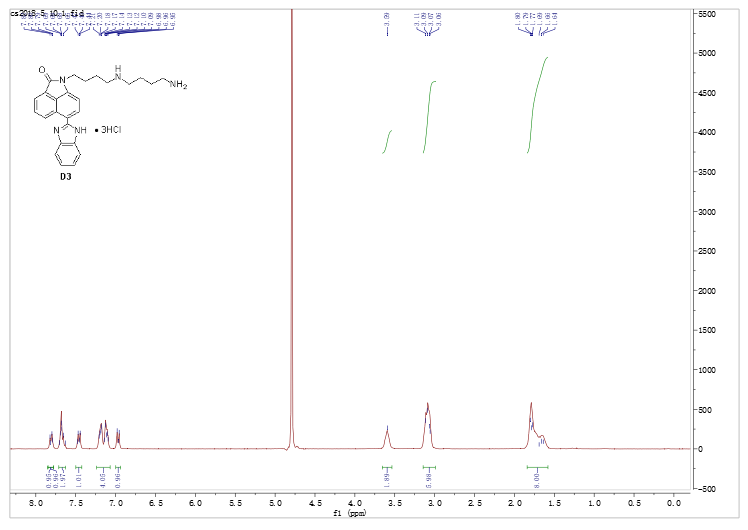
**

**
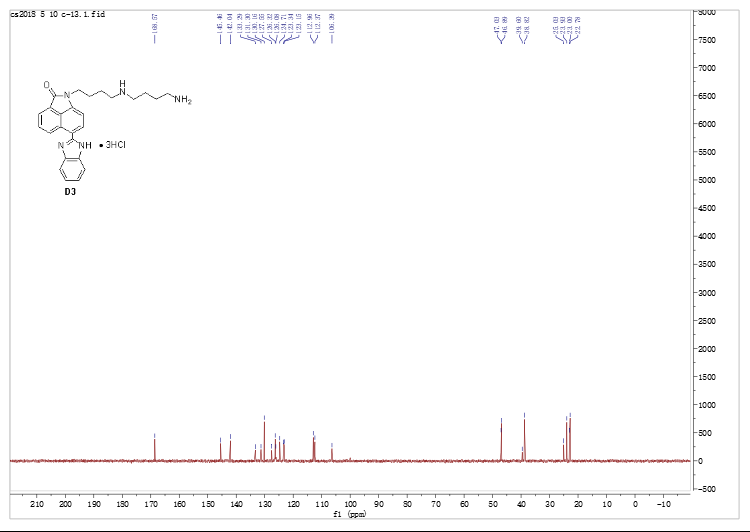
**

**
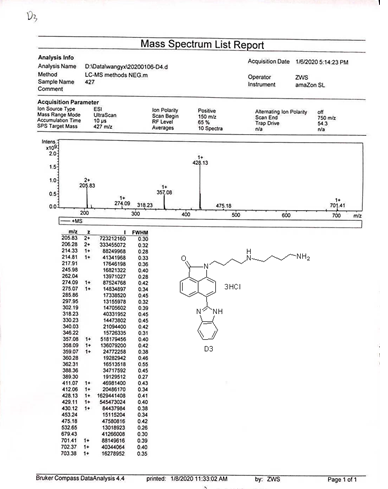
**
